# Combining docking, molecular dynamics simulations, AD-MET pharmacokinetics properties, and MMGBSA calculations to create specialized protocols for running effective virtual screening campaigns on the autoimmune disorder and SARS-CoV-2 main protease

**DOI:** 10.3389/fmolb.2023.1254230

**Published:** 2023-09-01

**Authors:** Emmanuel Israel Edache, Adamu Uzairu, Paul Andrew Mamza, Gideon Adamu Shallangwa, Fatma Hilal Yagin, Nagwan Abdel Samee, Noha F. Mahmoud

**Affiliations:** ^1^ Department of Pure and Applied Chemistry, University of Maiduguri, Maiduguri, Nigeria; ^2^ Department of Chemistry, Ahmadu Bello University, Zaria, Nigeria; ^3^ Department of Biostatistics and Medical Informatics, Faculty of Medicine, Inonu University, Malatya, Türkiye; ^4^ Department of Information Technology, College of Computer and Information Sciences, Princess Nourah bint Abdulrahman University, Riyadh, Saudi Arabia; ^5^ Rehabilitation Sciences Department, Health and Rehabilitation Sciences College, Princess Nourah bint Abdulrahman University, Riyadh, Saudi Arabia

**Keywords:** autoimmune disorder, type 1 diabetes, rheumatoid arthritis, SARS-CoV-2, CoMFA, docking, MD simulations

## Abstract

The development of novel medicines to treat autoimmune diseases and SARS-CoV-2 main protease (Mpro), a virus that can cause both acute and chronic illnesses, is an ongoing necessity for the global community. The primary objective of this research is to use CoMFA methods to evaluate the quantitative structure-activity relationship (QSAR) of a select group of chemicals concerning autoimmune illnesses. By performing a molecular docking analysis, we may verify previously observed tendencies and gain insight into how receptors and ligands interact. The results of the 3D QSAR models are quite satisfactory and give significant statistical results: Q_loo^∧^2 = 0.5548, Q_lto^∧^2 = 0.5278, R^∧^2 = 0.9990, F-test = 3,101.141, SDEC = 0.017 for the CoMFA FFDSEL, and Q_loo^∧^2 = 0.7033, Q_lto^∧^2 = 0.6827, Q_lmo^∧^2 = 0.6305, R^∧^2 = 0.9984, F-test = 1994.0374, SDEC = 0.0216 for CoMFA UVEPLS. The success of these two models in exceeding the external validation criteria used and adhering to the Tropsha and Glorbaikh criteria’s upper and lower bounds can be noted. We report the docking simulation of the compounds as an inhibitor of the SARS-CoV-2 Mpro and an autoimmune disorder in this context. For a few chosen autoimmune disorder receptors (protein tyrosine phosphatase, nonreceptor type 22 (lymphoid) isoform 1 (PTPN22), type 1 diabetes, rheumatoid arthritis, and SARS-CoV-2 Mpro, the optimal binding characteristics of the compounds were described. According to their potential for effectiveness, the studied compounds were ranked, and those that demonstrated higher molecular docking scores than the reference drugs were suggested as potential new drug candidates for the treatment of autoimmune disease and SARS-CoV-2 Mpro. Additionally, the results of analyses of drug similarity, ADME (Absorption, Distribution, Metabolism, and Excretion), and toxicity were used to screen the best-docked compounds in which compound 4 scaled through. Finally, molecular dynamics (MD) simulation was used to verify compound 4’s stability in the complex with the chosen autoimmune diseases and SARS-CoV-2 Mpro protein. This compound showed a steady trajectory and molecular characteristics with a predictable pattern of interactions. These findings suggest that compound 4 may hold potential as a therapy for autoimmune diseases and SARS-CoV-2 Mpro.

## 1 Introduction

Over 80 chronic, frequently life-threatening conditions in the family of autoimmune diseases were brought on by immune system deficiencies that caused the body to attack its tissues, organs, and cells (Fugger et al., 2020). Even though a lot of these illnesses are uncommon, they collectively affect 14.7 to 23.5 million people worldwide, and for unknown reasons, their prevalence is increasing (Sunagawa et al., 2020). Patients with the majority of autoimmune diseases must endure a lifetime of illness and treatment because there are currently no cures (Edache et al., 2022a). They frequently experience crippling symptoms, organ function loss, decreased productivity at work, and high medical costs (Elkhalifa et al., 2018). A significant burden is placed on patients’ families and society because most of these infections excessively influence ladies and are among the main sources of death for youthful and moderately aged ladies. Type 1 diabetes is one example of an autoimmune condition (DT1) (Reed and Herold, 2015). Among the most prevalent causes of death is diabetes. 463 million adults worldwide have diabetes as of 2019, claims the International Diabetes Federation (IDF) (International Diabetes Federation, 2019; Andalia et al., 2022). That number is anticipated to increase to about 700 million by 2045 (International Federation Federation, 2019). 79% of diabetes-affected adults reside in low- and middle-class nations. In Nigeria, there were 1,702,900 diabetes cases in 2015 (Rahamon, 2020). With 3.9 million diabetics, Nigeria had the highest prevalence as of 2016. By 2045, this amount will have doubled. Diabetes in Nigeria was not well understood in the 1990s. Today, diabetes is a concern for the typical household (International Federation Federation, 2019; Rahamon, 2020). Diabetes is the primary factor in many serious illnesses, including heart failure, cardiovascular conditions like stroke, sexual dysfunction, nephropathy, retinopathy, vascular dysfunction, blindness, and various cancers (Jassim et al., 2021). Most diabetic patients experience non-healing wounds, which can result in the amputation of hands, feet, and other body parts. Additionally, the main risk factor for chronic kidney disease is diabetes (International Diabetes Federation, 2019; International Federation Federation, 2019; Rahamon, 2020; Jassim et al., 2021). Nigeria’s healthcare system is among the worst in the world, and its poverty rate is extremely low (Jassim et al., 2021). Rheumatoid arthritis (RA) is yet another condition that is autoimmune in nature (Edache et al., 2022a). One of the most inflammatory illnesses is RA. It is a long-lasting autoimmune condition that causes symmetrical and bilateral joint inflammation (Khither et al., 2020). Symmetrical, multiple-joint inflammatory lesions that have been present for a while dominate the clinical picture of RA (Zhou et al., 2019; Edache et al., 2022a). As the disease progresses, other body organs and systems may also be impacted, including the eyes, heart, lungs, kidneys, physical fitness, and other internal organs (Derksen et al., 2017; Oh et al., 2022). As a result of joint inflammation, RA typically causes fever and swollen, and painful joints (Zhou et al., 2019). The actual causes and mechanisms triggering the onset and progression of rheumatoid arthritis (RA) are not well understood (Lin et al., 2020). But it is understood that this dysfunction is characterized by a long-lasting autoimmune condition that primarily affects the synovial joint lining (Mrid et al., 2022). As of 2018, the World Health Organization estimates that RA affected more than 30 million people worldwide, with an average occurrence of 0.5%–1% among adults (Heijde et al., 2018). Teenagers, adults, and seniors can develop it, however, women around the ages of 40 and 60 make up the majority of patients (Zhou et al., 2020). Acute respiratory distress syndrome (ARDS) and cytokine storm may be linked to serious outcomes for patients with autoimmune disorders who are more vulnerable to viral infections (Yang, 2021).

The virus can damage organ tissue and cause multiple organ dysfunction syndrome by infecting cells in the lungs, kidneys, heart, and intestine (Wu et al., 2020). Direct interaction with people and facial matter may involve in the transmission of SARS-CoV-2, which is primarily spread through respiratory droplets (Edache et al., 2022b). SARS-CoV-2 primarily affects the respiratory tract, starting with the symptoms of a cold, fever, dry cough, exhaustion, sore throat, and diarrhea and progressing to severe pneumonia, breathing difficulties, and patient death (Zhou et al., 2020). Risk factors for viral infection include DT1 and RA. According to Alqahtani et al. (2020), older adults with chronic comorbidities like diabetes mellitus have been identified as having the most severe SARS-CoV-2 cases. According to a team of researchers, 68% of MERS patients also had an autoimmune disease, such as diabetes (Assiri et al., 2013). Diabetes has been linked to a higher risk of MERS, according to particular circumstance research (Alraddadi et al., 2014). According to Yang et al. (2017), autoimmune diseases like diabetes have also been linked to a higher mortality rate in MERS patients. Diabetes has been linked to immune responses that are dysregulated in animal studies, which leads to lung pathology that is more severe and lasts longer after MERS-CoV infection (Kulcsar et al., 2019). In comparison to patients without autoimmune disorders, patients with autoimmune diseases (DT1 and RA) and viral SARI would experience a challenging illness with far worse consequences.

There is currently no effective treatment for autoimmune disorders (DT1 and RA), and those that are used aim to lessen joint inflammation, stop irreversible bone loss, and keep joints functioning as much as possible (Guo et al., 2018). Patients with an autoimmune disorder are tired of a regular lifestyle, tired of feeding like they are a piece of an experiment, and also sick of drugs day in and day out without signs of improvement. Lymphoid tyrosine phosphatase (LYP) encoded by the gene PTPN22 has been found to increase the risk of many autoimmune diseases (Lee et al., 2007; Ban et al., 2010). The protein tyrosine phosphatase LYP, which is specific to lymphocytes, is essential for controlling T cell receptor activation (Rieck et al., 2007; Ban et al., 2010). The PTPN22 gene encodes this phosphatase. The autoimmunity predisposing PTPN22 is a gain-of-function mutant suggesting that a specific small-molecule inhibitor could eliminate its effect. In the creation of new SARS-CoV-2 inhibitors, the main protease (Mpro), which has an important function in the virus mitotic phase, has been regarded as a possible target. The substantial role of PTPN22 and Mpro makes it an attractive target for developing anti-autoimmune disorders and SARS-CoV-2 agents (Edache et al., 2022a; Edache et al., 2022c).

The process of creating a drug molecule is iterative, starting with a lead molecule with an ideal living organism’s trait and concluding with its improvement, leading to the choice of a candidate molecule for drug development (Adedirin et al., 2018). The process of discovering new drugs is extremely complicated and involves an interdisciplinary effort to develop medicines that are both efficient and marketable (Gurung et al., 2021). A computer is incredibly important in pharmaceutical, medical, and other scientific research, even in the creation of novel compounds in the search for more effective therapeutic agents. To find novel therapeutic agents, structural biology and rational drug design are combined. Using computer-aided drug design (CADD) also referred to as *in silico* techniques, is one way to improve the efficacy of developing new drugs. These methods take a computational chemistry approach to the process of finding new drugs (Maia et al., 2020) To find new chemical entities, the CADD center collaborates with structure biologists, biophysicists, and computational scientists. Tools like CADD and bioinformatics can help accelerate drug search and production while also saving money, reducing time to market, and learning more about how drugs interact with their receptors. Every drug undergoes a lengthy development and discovery process that starts with scientific research on the disease, identification of target receptors, selection of active compounds from a large pool of compounds, etc. CADD is now a crucial tool for accelerating the development of posttranscriptional inhibitors by assisting with compound collection, design, and lead identification.

For this study, a group of 31 carefully chosen compounds with potent and specific affinities for autoimmune disorders [https://pubchem.ncbi.nlm.nih.gov/bioassay/435024] were chosen to determine a detailed connection between their organizational structures, interactions, and activities. To find new molecules that are effective against autoimmune disorders and the SARS-CoV-2 virus, several computational techniques have been used, including molecular docking and the analysis of dynamics interaction. To ensure the validity of the 3D-QSAR analysis, the precise binding modes of the selected compounds against autoimmune disorder and the SARS-coronavirus-2 virus were examined through docking simulations and molecular dynamics simulations. This research is anticipated to offer a theoretical direction for the investigation, forecasting, and creation of novel agonists to treat autoimmune disorders and the SARS-coronavirus-2 virus.

## 2 Materials and methods

The molecular structure of the selected molecules, as depicted in Table 1, obtained from the PubChem database (AID 435024) was pre-optimized with Avogadro v1.2 software (Hanwell et al., 2012) and then optimized to standard convergence criteria by semi-empirical method with MOPAC v22.0.4 (Stewart, 2013).

**TABLE 1:** Retrieval of chemical compounds from PubChem Database.

S/N	PUBCHEM_CID	Compound name	pIC50
1	647,501	1-ethyl-6-methyl-3-phenyl-1H,5H,6H,7H-pyrimido [5,4-e][1,2,4]triazine-5,7-dione	4.9821
2	654,089	(3aR,4S,9bS)-6-hydroxy-3H,3aH,4H,5H,9bH-cyclopenta [c]quinoline-4-carboxylic acid	5.0783
3	573,747	3,4-bis(thiophene-2-carbonyl)-2,3-dihydro-1,2,5-oxadiazol-2-ol	5.9066
4	3,239,469	4-[(12-{1,4-dioxa-8-azaspiro [4.5]decan-8-yl}-8-oxo-15-oxa-14-azatetracyclo [7.6.1.0^2^,⁷.0^1^³,^1^⁶]hexadeca-1(16),2(7),3,5,9,11,13-heptaen-10-yl)amino]butanoic acid	4.208
5	66,541	1,6-dimethylpyrimido [5,4-e][1,2,4]triazine-5,7-dione	5.9066
6	460,747	1,3,6-trimethyl-1H,2H,5H,6H,7H,8H-pyrimido [5,4-e][1,2,4]triazine-5,7-dione	5.9066
7	1,973,720	3-[(2-hydroxyethyl)dimethylamino]-N-[2-methyl-1-(trihydroxy-λ⁴-sulfanyl)propan-2-yl]propanamide	5.3898
8	2,012,947	4-[(5Z)-5-{[5-(1,3-benzothiazol-2-yl)furan-2-yl]methylidene}-4-oxo-2-sulfanylidene-1,3-thiazolidin-3-yl]butanoic acid	5.3212
9	3,116,376	(1S,2S,3aS,4R,9bS)-8-acetyl-1-chloro-2-[(2-nitrophenyl)sulfanyl]-1H,2H,3H,3aH,4H,5H,9bH-cyclopenta [c]quinoline-4-carboxylic acid	4.2377
10	1,714,876	N-{4-[(5-ethyl-1,3,4-thiadiazol-2-yl)sulfamoyl]phenyl}-2-oxo-8-(prop-2-en-1-yl)-2H-chromene-3-carboxamide	5.0256
11	86,261,486	N'-[(E)-(2-hydroxy-3-methoxyphenyl)methylidene]-5-nitro-1-benzothiophene-2-carbohydrazide	4.8825
12	1,334,608	2-{[5-(ethoxycarbonyl)-12-(2-hydroxybenzoyl)-4-methyl-2-oxo-6-thia-1,8-diazatricyclo [7.4.0.0³,⁷]trideca-3 (7),4,8,10,12-pentaen-10-yl]sulfanyl}acetic acid	5.6209
13	9,564,046	4-[(2-bromo-4-{[(4Z)-2,5-dioxoimidazolidin-4-ylidene]methyl}-6-ethoxyphenoxy)methyl]benzoic acid	4.4251
14	9,595,043	2-{4-[(E)-{[(4-hydroxyphenyl)formamido]imino}methyl]phenoxy}-N-(3-nitrophenyl)acetamide	5.3295
15	5,995,173	2-{2-ethoxy-4-[(E)-{[(4-{2-[(4-methylphenyl)amino]-1,3-thiazol-4-yl}phenyl)formamido]imino}methyl]phenoxy}acetic acid	5.9066
16	2,545,524	2-(4-{[(2Z,5Z)-3-[2-(1H-indol-3-yl)ethyl]-2-[(4-methoxyphenyl)imino]-4-oxo-1,3-thiazolidin-5-ylidene]methyl}phenoxy)acetic acid	5.6946
17	2,975,144	3-{[5-(ethoxycarbonyl)-12-(2-hydroxy-5-methoxybenzoyl)-4-methyl-2-oxo-6-thia-1,8-diazatricyclo [7.4.0.0³,⁷]trideca-3 (7),4,8,10,12-pentaen-10-yl]sulfanyl}propanoic acid	4.314
18	2,229,326	4-[(3aR,4R,9bS)-8-[(3-chloro-2-methylphenyl)sulfamoyl]-3H,3aH,4H,5H,9bH-cyclopenta [c]quinolin-4-yl]benzoic acid	4.8054
19	5,756,371	4-({2-bromo-4-[(1E)-2-cyano-2-(3-fluorophenyl)eth-1-en-1-yl]phenoxy}methyl)benzoic acid	4.9317
20	3,164,059	1-ethyl-6-methyl-3-[(1E)-2-phenylethenyl]-1H,5H,6H,7H-pyrimido [5,4-e][1,2,4]triazine-5,7-dione	5.7964
21	7,217,786	1,6-dimethyl-3-propyl-1H,5H,6H,7H-pyrimido [5,4-e][1,2,4]triazine-5,7-dione	4.415
22	9,595,032	5-chloro-2-methoxy-N-(3-{[1,2,4]triazolo [4,3-b]pyridazin-6-yl}phenyl)benzamide	5.9066
23	25,250,764	2-{4-[(E)-{[(4-{2-[(4-chlorophenyl)amino]-1,3-thiazol-4-yl}phenyl)formamido]imino}methyl]phenoxy}acetic acid	5.0665
24	6,104,167	4-(3-{[(E)-N'-[(E)-[(2H-1,3-benzodioxol-5-yl)methylidene]amino]carbamimidoyl]sulfanyl}-2,5-dioxopyrrolidin-1-yl)benzoic acid	5.3562
25	1,587,127	3-(2-{[(4E)-1-(4-chlorophenyl)-2,5-dioxoimidazolidin-4-ylidene]methyl}-1H-pyrrol-1-yl)benzoic acid	5.5599
26	1,516,220	4-{3-[(5Z)-5-[(3,4-dimethoxyphenyl)methylidene]-4-oxo-2-sulfanylidene-1,3-thiazolidin-3-yl]propanamido}benzoic acid	5.1922
27	8,853,383	3-[(2E)-3-{4-[(4-chlorophenyl)methoxy]phenyl}-2-cyanoprop-2-enamido]benzoic acid	4.9512
28	2,354,598	5-methyl-2-[(1E)-2-(4-methyl-3-nitrophenyl)ethenyl]-4-oxo-3H,4H-thieno [2,3-d]pyrimidine-6-carboxylic acid	5.9066
29	2,867,365	3-[(2E)-3-{4-[(4-bromophenyl)methoxy]-3-ethoxyphenyl}-2-cyanoprop-2-enamido]benzoic acid	4.8247
30	1,889,464	4-methyl-3-[2-(4-nitrophenyl)-1,3-dioxo-2,3-dihydro-1H-isoindole-5-amido]benzoic acid	4.8604
31	2,545,467	3-{5-[(3Z)-1-{[(4-methoxyphenyl)carbamoyl]methyl}-2-oxo-2,3-dihydro-1H-indol-3-ylidene]-4-oxo-2-sulfanylidene-1,3-thiazolidin-3-yl}propanoic acid	5.9066

### 2.1 Molecular modeling for 3D-QSAR (Alignment and CoMFA analysis)

**FIGURE 1:**
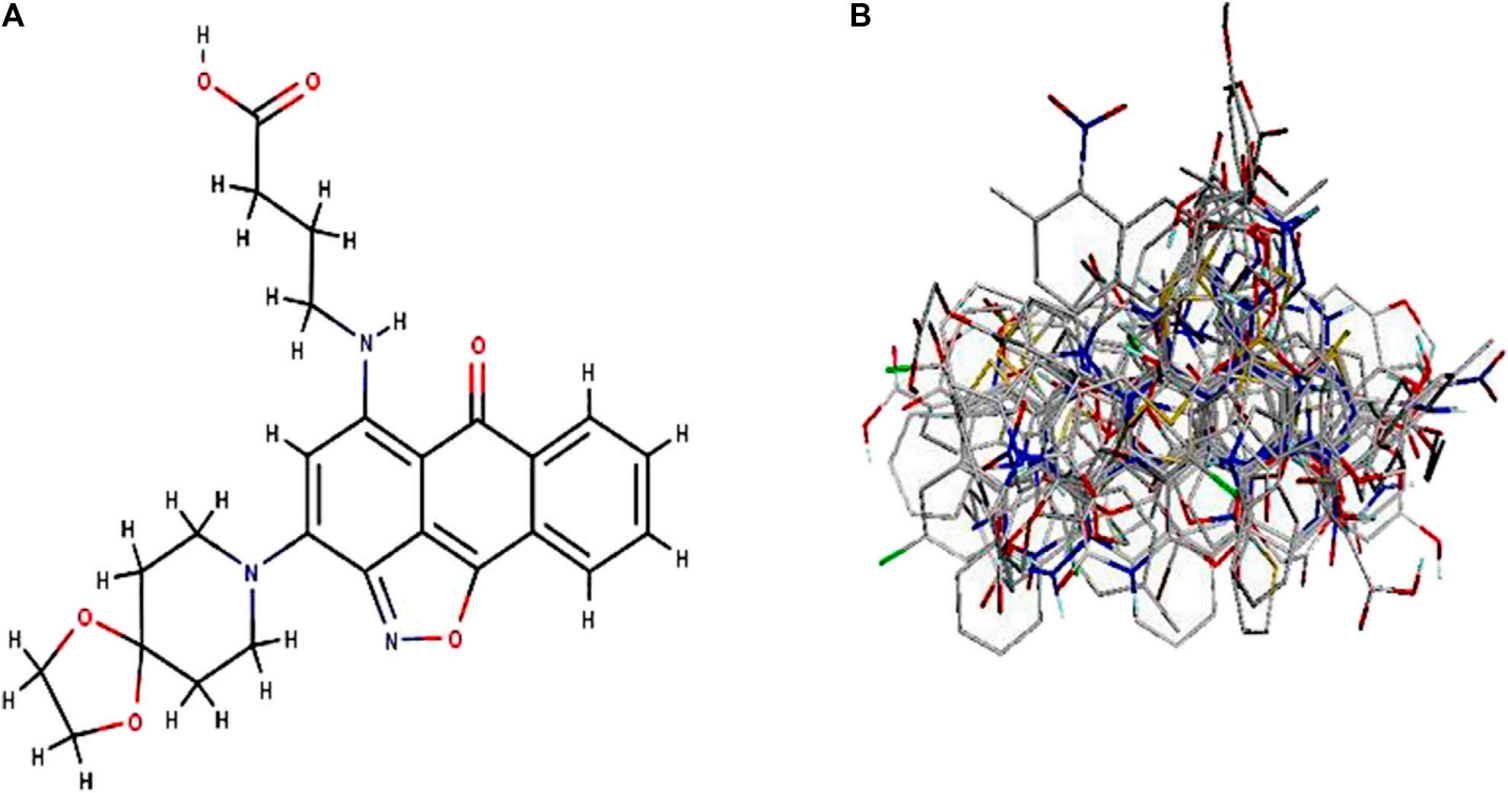
3D modeling of Compound 4 and its alignment. Part **(A)**: Compound 4 structure was used as a template 513 for docking-based alignment; Part **(B)**: the alignment for the autoimmune disorder was derived from 514 the docking-based alignment obtained from Open3DALIGN.

By taking, the observed IC50 values of all compounds in M were converted into pIC50 = -Log (1/IC50), which was then used as the dependent variable. For the categorization of the compounds into training and test sets in this research, QSARINS v2.2.4 (Gramatica et al., 2013) was used in the most random manner possible. The data set has a homogenous distribution and includes two sets: a training set (20 compounds, 70%) and a test set (11 compounds, 30%). The PubChem CID number, IUPAC name, and inhibitory activities were listed in Table 1. The open3DALIGN tools’ docking-based alignment was used to superimpose the compounds (Tosco et al., 2011). Compound 4 was chosen as the format to line up other compounds because of its strong autodocking rating and the alignment was finished by the open3DALIGN software as depicted in Figure 1. The calculation of molecular field descriptors was then applied to the compound alignment.

The electrostatic field was determined utilizing a volume-less +1 charge test (Tosco and Balle, 2011), though the steric field was determined utilizing a carbon molecule test for the Open3DQSAR programming (Kumar et al., 2015). Some erroneous variances were eliminated to shrink the partial least square (PLS) framework. As part of the data pre-treatment operation, additional N-level variable removal was carried out (Kumar et al., 2015). These N-level variables posit a distribution of only N values across a constrained number of training set objects. The Smart Region Definition (SRD) algorithm, which is rooted in factor connectedness in 3D space, was then used to group the variables (Pastor et al., 1997). Then, a variety of variable selection techniques, such as Fractional Factorial Design (FFDSEL) and Uninformative Variable Elimination-Partial Least Square (UVE-PLS) variable selection (Baroni et al., 1992; Centner et al., 1996), were used to develop the best PLS models. Cross-validated LOO (Leave-One-Out), LTO (Leave-Two-Out), or LMO (Leave-Many-Out) paradigms were used to compute PLS models (Tosco and Balle, 2011; Abdel Samee et al., 2012; Abdel Samee, 2020; Nawaz et al., 2022). Last but not least, the Maestro graphics package was used to visualize PLS coefficient grid maps or the activity-correlating molecular regions in the form of iso-contour maps.

### 2.2 Protein setup

For the present study, two different crystallized structures of type 1 diabetes (PDB ID: 1JK8 and 1XW7), rheumatoid arthritis (PDB ID: 2AXJ and 2FSE), one for protein tyrosine phosphatase, nonreceptor type 22 (lymphoid) isoform 1 (PTPN22) (PDB ID: 4J51), and one for SARS-CoV-2 main protease (PDB ID: 6LU7) were used to test the validity of Autodocking vina docking program with Assisted Molecular Docking with AutoDock4 and Autodocking Vina (AMDock) v1.5.2 (Valdés-Tresanco et al., 2020).

### 2.3 Molecular docking

Molecular docking simulations, as a right approach, were employed using the AMDock v1.5.2 (Valdés-Tresanco et al., 2020) to discover the structural interaction mechanism between ligands and the diabetes type 1, rheumatoid arthritis, and SARS-CoV-2 main protease receptors. The 3D crystallized structure of diabetes type 1, rheumatoid arthritis, PTPN22, and SARS-CoV-2 main protease receptors was made available by Protein Data Bank. To create docking simulations, the docking rule’s setup procedures were used. The co-crystallized ligand and all water molecules were separated from the protein using the Discovery Studio client software, and the binding modes were calculated using Optimal box size 1.1 (Feinstein and Brylinski, 2015) and AutoLigand (Harris et al., 2008) embedded in AMDock software. The AMDock experimental tool with the autodocking Vina method (Trott and Olson, 2010) was employed to find the best-docked ligands with the receptors. The Discovery Studio 2020 client software was used to analyze the results of the studies of ligand-protein interactions. The results of the docking were represented by the binding affinity scores as affinity/ ∆G (Kcal/mol), and they were further converted to the estimated inhibition constants (Ki). The following formula was used to calculate the Ki parameters from the binding affinity values for each docked pose:

$$K_{I}=\exp^{\left(\frac{∆G}{RT}\right)}$$ (1)

where ΔG is the binding affinity or the calculated docking score value, R was Boltzmann gas constant (= 1.987 cal/mol/K), and T was the temperature (= 298 K), respectively.

In comparison to the affinity, the estimated Ki is a much more useful quality because it is more closely related to frequently measured experimental parameters. On the other hand, ligand efficiency (LE) is a crucial informative factor when choosing a lead compound (Kenny, 2019). The following equation is used to determine LE in this situation:

$$LE=\left(-∆G\right)/HA$$ (2)

where HA is the ligand’s total number of heavy (non-hydrogen) atoms. Potential lead compounds are indicated by compounds with LE 0.3 (Schultes et al., 2010). For additional molecular dynamics simulations, the receptor-ligand binding conformation with the largest negative docking score (binding affinity) was examined.

### 2.4 Molecular dynamics (MD) simulation

To assess the stability, Molecular Dynamics (MD) simulations were run and probed the dynamic conformational changes of the selected complexes using Nano Scale Molecular Dynamics (NAMD v2.14) software (Phillips et al., 2005). The receptor-ligand complex obtained from molecular docking simulations was the initial structure used for MD simulations. In CHARMM-GUI, the topologies and parameter files for the complexes (protein-ligand) were generated using Solution Builder (Jo et al., 2008; Jo et al., 2014; Lee et al., 2016; Kim et al., 2017). The system is solvated by adding the TIP3P model to the solvation box, and the counter ions (NaCl 0.15M) were used to neutralize the simulation box. The complex is then subjected to the CHARMM36m force field (Huang et al., 2016) at a constant number of molecules, volume, and temperature (NVT). The steepest descent algorithm was used to complete 20,000 steps of energy minimization. The energy-minimized frameworks were then put to use for simulations at a constant number of molecules, pressure, and temperature (NPT) using Langevin dynamics parameters, the temperature at 310K, and for 10 ns under constant Periodic Boundary conditions to compare trajectories. The MD trajectories were analyzed using the script in the VMD v1.9.3 software (Humphrey et al., 1996), including root mean square deviation (RMSD), root mean square variation (RMSF), solvent accessible surface area (SASA), and radius of gyration (Rg).

### 2.5 Binding free energy calculation (MM-GBSA)

The Molecular Mechanics/General-Boltzmann Surface Area (MM-GBSA) method was performed to estimate the binding free energy ( ${∆G}_{binding}$ ) of the protein-ligand complex, which is a popular endpoint technique for calculating free energy (Wang et al., 2019). Using MolAlCal (Bai et al., 2021), the binding free energies of the complex in liquid/solution were calculated by Eq. 3:

$${\delta G}_{bind}={\delta G}_{complex}-\left({\delta G}_{protein}+{\delta G}_{ligand}\right)$$ (3)

where, ${\delta G}_{bind}$ , ${\delta G}_{complex}$ , ${\delta G}_{protein}$ , and ${\delta G}_{ligand}$ are calculated free binding energy, calculated free binding of the complex, calculated free binding of the protein, and calculated free binding energy of the ligand, respectively.

### 2.6 Prognostication of ADMET by computational analysis

Drug research time has been significantly shortened in the past few years by the creation of computerized (*in silico*) modeling techniques to evaluate absorption, distribution, metabolism, excretion, and toxicity (ADMET) attributes. It is easier to exclude compounds with prospective ADMET issues when these attributes can be predicted quickly and accurately. This aids researchers in making decisions about which compounds to generate and test first (Mary et al., 2021)

## 3 Results and discussion

3D-QSAR models were created using autoimmune disease inhibitors. Table 2 summarizes the findings of the CoMFA [fractional factorial design (FFDSEL) and uninformative variable elimination-partial least square (UVEPLS)] studies. The values for the R^2, F-test, SDEC, Q_loo^2, Q_l2o^2, Q_lmo^2, and SDEP were calculated according to the PLS analysis definitions. The CoMFA FFDSEL analysis Q_l2o^2 value of 0.5278, and a Q_loo^2 value of 0.5548 respectively. The fitting PLS analysis yields a coefficient of determination R^2 of 0.9990, F-test = 3101.1411, and a standard deviation of the error of training set predictions (SDEC) of 0.0173 for CoMFA FFDSEL analysis. The commitments from steric and electrostatic forces were, 44.93% and 55.07%, respectively. These findings suggest that the binding affinity was primarily influenced by the electrostatic field.

**TABLE 2:** Computed data for the CoMFA (FFDSEL and UVEPLS) models.

Model	$R^{2}$	F−test	SDEC	$Q_{loo}^{2}$	${SDEP}_{loo}$	$Q_{l2o}^{2}$	${SDEP}_{l2o}$	$Q_{lmo}^{2}$	${SDEP}_{lmo}\pm SD$
CoMFA (FFDSEL)	0.9990	3,101.1411	0.0173	0.5548	0.3604	0.5278	0.3712	0.4721	0.391 ± 0.0336
CoMFA (UVEPLS)	0.9984	1994.0374	0.0216	0.7033	0.2942	0.6827	0.3043	0.6305	0.3276 ± 0.0224

The CoMFA (UVEPLS) model was created by combining the steric and electrostatic fields; the outcomes are (Q_loo^2 = 0.7033, Q_lto^2 = 0.6827, Q_lmo^2 = 0.6305, R^2 = 0.9984, F-test = 1994.0374, SDEC = 0.0216) from combining these 2 fields with the 5 components. The corresponding field contributions are 54.58% (steric) and 45.42% (electrostatic), this indicates that the steric of the molecule affects its ability to act as an inhibitor The higher worth of the F-test, the more prominent the likelihood that the 3D-QSAR model is considerable. The F-test measures for the CoMFA (FFDSEL) and CoMFA (UVEPLS) models were 3101.1411 and 1994.0374 respectively. The level of statistical confidence is represented by the F-test value. Table 3 compares predicted and actual pIC50 values for CoMFA, and their residues (for the training and test sets).

**TABLE 3:** Actual and predicted pIC50 for Autoimmune disease inhibitors of training and test set for the CoMFA (FFDSEL and UVEPLS) models.

Cpd No.	pIC50	FFDSEL	Residues	UVEPLS	Residues
1	4.9821	5.0353	0.0532	5.0368	0.0547
2	5.0783	5.0828	0.0045	5.1028	0.0245
3	5.9066	5.8914	−0.0152	5.8623	−0.0443
4	4.208	4.2022	−0.0058	4.1777	−0.0303
5^T^	5.9066	5.6841	−0.2225	5.7253	−0.1813
6	5.9066	5.9134	0.0068	5.9163	0.0097
7^T^	5.3898	5.4963	0.1065	5.3324	−0.0574
8^T^	5.3212	5.0325	−0.2887	4.9931	−0.3281
9^T^	4.2377	4.7993	0.5616	4.7328	0.4951
10	5.0256	5.032	0.0064	5.0316	0.006
1^T^	4.8825	4.9605	0.078	4.991	0.1085
12	5.6209	5.6058	−0.0151	5.6225	0.0016
13	4.4251	4.4022	−0.0229	4.4206	−0.0045
14^T^	5.3295	5.1948	−0.1347	5.0565	−0.273
15	5.9066	5.9134	0.0068	5.9018	−0.0048
16	5.6946	5.7065	0.0119	5.7177	0.0231
17	4.314	4.316	0.002	4.3208	0.0068
18	4.8054	4.8019	−0.0035	4.8041	−0.0013
19	4.9317	4.9092	−0.0225	4.9552	0.0235
20	5.7964	5.7713	−0.0251	5.7755	−0.0209
21^T^	4.415	5.4392	1.0242	5.4792	1.0642
22^T^	5.9066	5.1667	−0.7399	5.2997	−0.6069
23	5.0665	5.0541	−0.0124	5.0475	−0.019
24^T^	5.3562	5.8161	0.4599	5.8035	0.4473
25	5.5599	5.5712	0.0113	5.5696	0.0097
26	5.1922	5.1893	−0.0029	5.1659	−0.0263
27	4.9512	4.9748	0.0236	4.9329	−0.0183
28	5.9066	5.9155	0.0089	5.9257	0.0191
29	4.8247	4.8182	−0.0065	4.8278	0.0031
30	4.8604	4.8719	0.0115	4.8626	0.0022
31	5.9066	5.8916	−0.015	5.8922	−0.0144

T Test set compounds.

**FIGURE 2:**
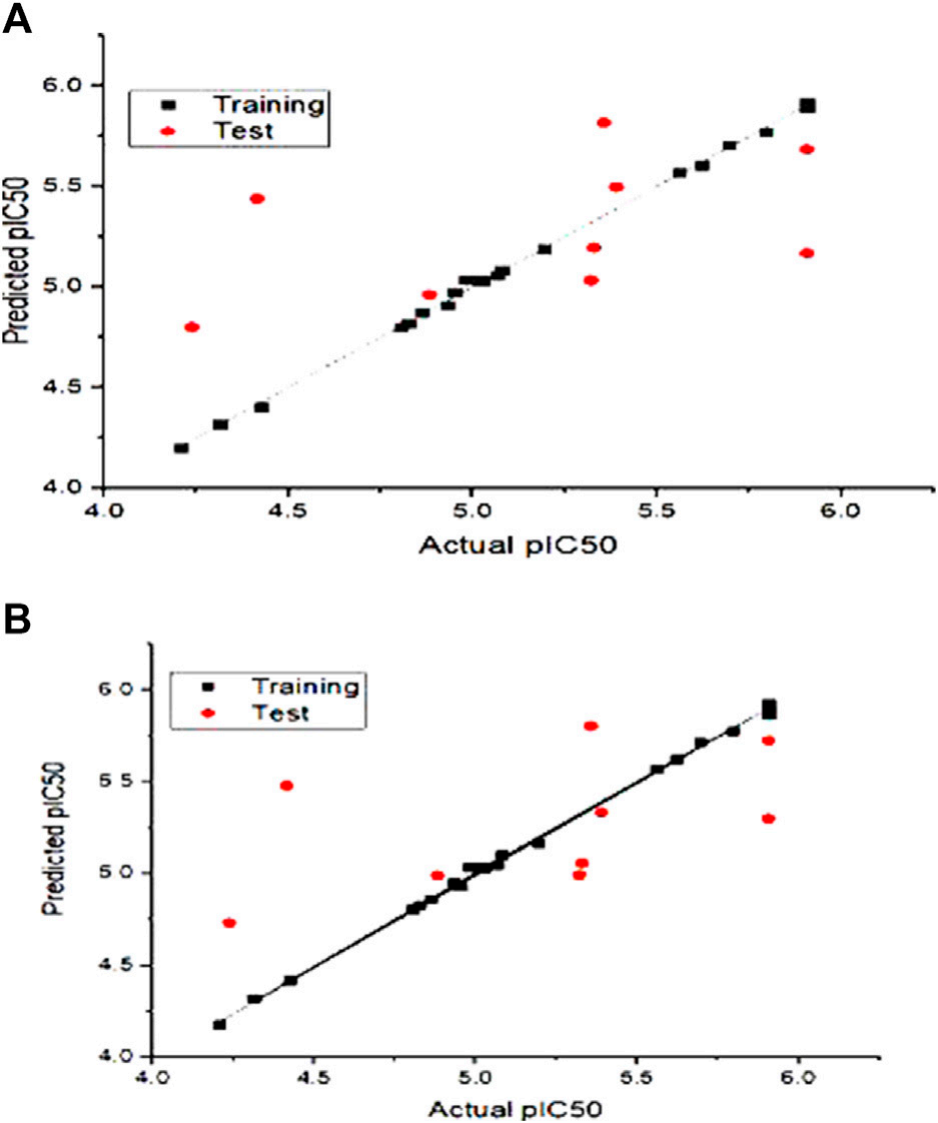
Graph of autoimmune disease inhibitors predicted pIC50 of training and test set from CoMFa **(A)** FFDSEL and **(B)** UVEPLs analysis.

The actual vs. the predicted activities for various compounds are also displayed in Figure 2, supporting the Open3DQSAR model’s superior predictive power. The activities predicted by the CoMFA model, and the experimental data agree well, as shown in Figure 2, suggesting that the CoMFA model has respectable predictive power. The CoMFA FFDSEL and UVEPLS models show a small statistical difference, indicating that the two fields contribute nearly equal amounts to the relationship.

### 3.1 Analysis of the contour map

**FIGURE 3:**
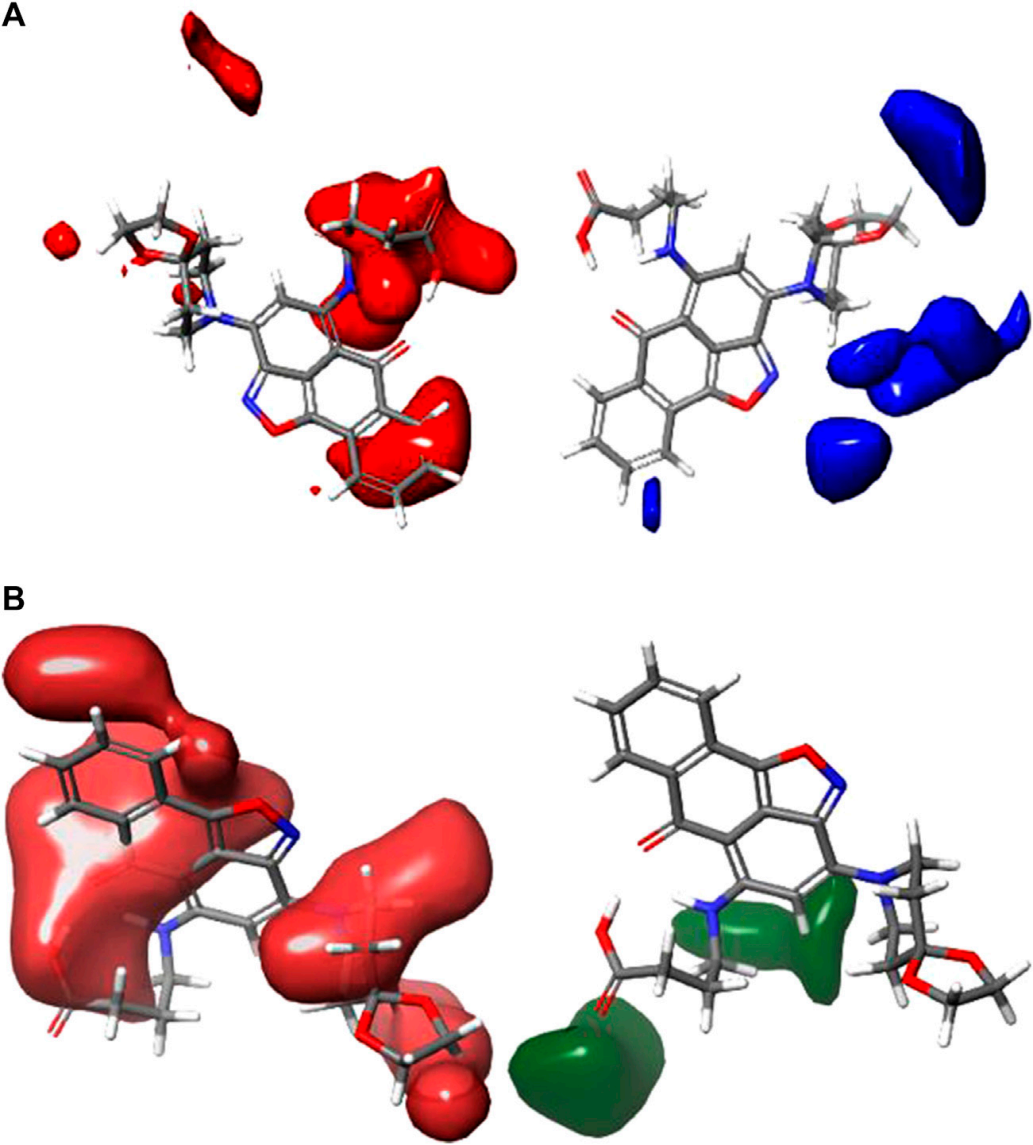
Shape guides of CoMFA FFDSEL: **(A)** steric feild and **(B)** electronstatic field in view of comound 4.

CoMFA figure plots were created to depict the regions in 3D space around the compound where changes in the steric and electrostatic fields were predicted to increase or decrease activity to speculate about the information content of the derived 3D QSAR model. A full assessment of the obtained contours identifies the crucial physicochemical factors governing the activity and explores the crucial role played by the complex formation in their 3D orientation. The CoMFA contour map was created using Compound 4 as the reference structure. Figure 3A depicts the steric contour map for the CoMFA FFDSEL model. Areas, where the large groups do not encourage activity, are indicated by red contour maps (negative region), while these areas that encourage large groups are indicated by blue contour maps (positive region). Electrostatic interactions are depicted in Figure 3B by contours in maroon and green colors. Green regions only increase activity with negative charges, while maroon regions indicate that positive charges are preferred. To generate higher an-ti-autoimmune disease activity, a bulky or large group is not preferred, according to the region of the steric contour map with the large percentage of red color. For instance, due to fewer bulky groups in that area, the other compounds have higher anti-autoimmune disease activity than compound 4. The reference compound (compound 4) is surrounded by a maroon region on the electrostatic contour map, which suggests that the activity of the electron-withdrawing substituent may be improved. The anti-autoimmune disease activity of certain compounds is found to be increased by an electron-withdrawing group (such as 9, 13, 19, 22, 25, 27, and 29). The involvement of an electron-rich substituent is advantageous at this position, as shown by the green electrostatic contour surrounding the reference compound 4. It suggests that alkoxy groups, such as -OCH3, are preferred in that position over the -CO group because they are more electron-rich and easily accept donated electrons while being incapable of ionization.

**FIGURE 4:**
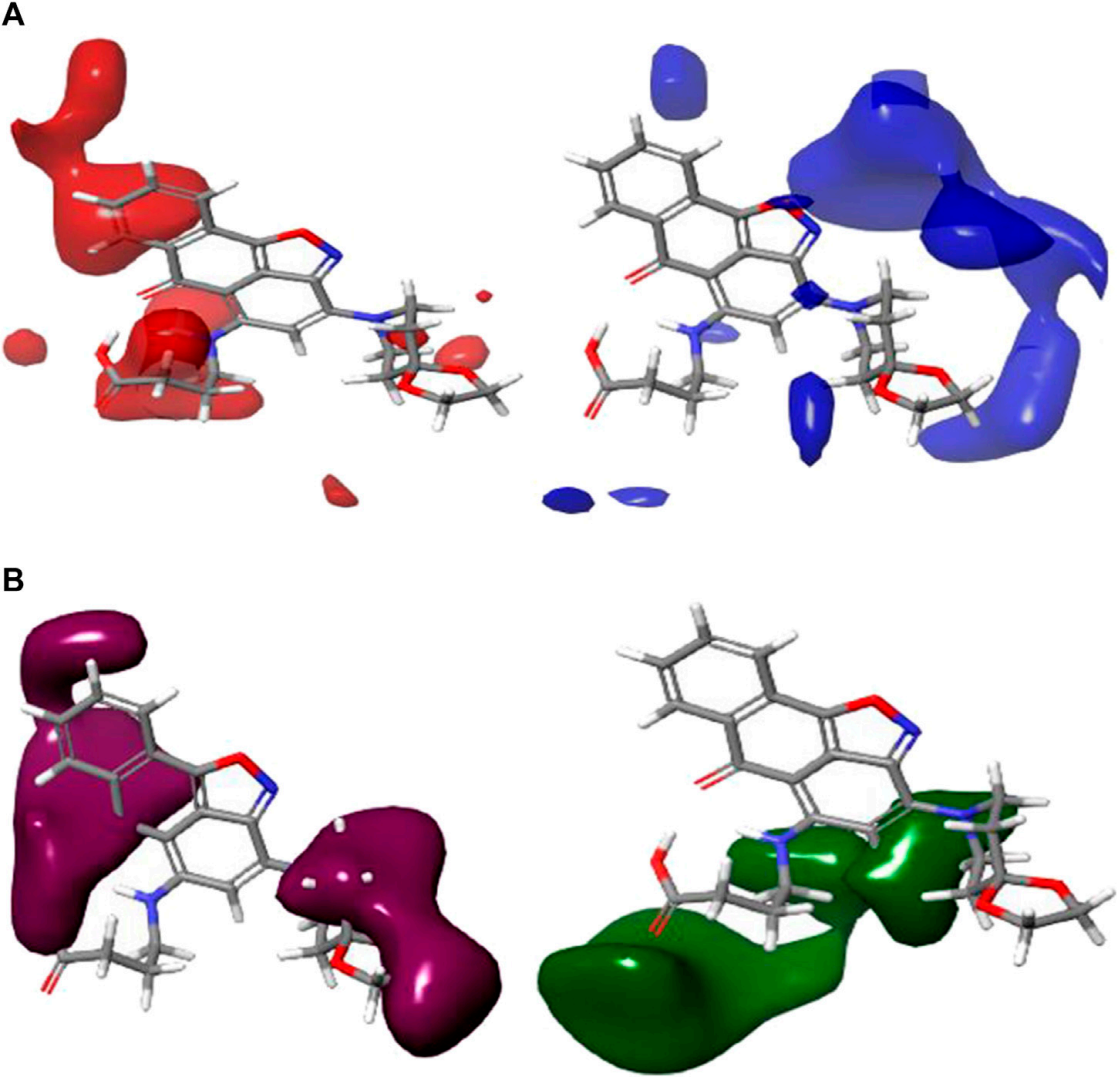
Charts of CoMFA UVEPLS **(A)** steric field and **(B)** electrostatic field grounded on compound 4.

The 3D contour maps created using the CoMFA UVEPLS model to suggest a 3D-QSAR model on the target are shown in Figure 4. Red and blue contours stand in for steric interactions, while maroon and green contours stand for electrostatic interactions. The blue contours in this diagram indicate the areas where the involvement of bulkier groups (53% contribution) would contribute to improving biological activity, while the red contours (47% contribution) show the areas where such bulkier groups have the opposite effect and cause biological activity to decrease. The influences of the steric field are shown in Figure 4A. The maroon and green contours on the CoMFA UVE-PLS electrostatic contour maps are depicted in Figure 4B. The blue contours (44% contribution) show the regions where the involvement of electron-rich (electronegative) groups would enhance biochemical activity, while the maroon contours (contribution of 56%) show the areas where the positively charged (or reduced negatively charged) group leads to an increase in biochemical activity.

### 3.2 Docking analysis and FDA-approved drugs (standards)

The thirty-one selected compounds and some FDA-approved drugs (as standard) were studied *in silico* to select the least binding affinity with the active site of some autoimmune diseases and SARS-coronavirus-2 Mpro, using the AMDock software with AD-Vina algorithm. The docking scores, estimated inhibition constants (K_i), and ligand efficiency (LE) of the binding site of all the receptors used in these studies are summarized in Supplementary Table S1. At first, to choose the best hit-lead compound, the binding affinity of the PTPN22 receptor was used as a criterion to select the least docking score. Based on the findings of the docking screening, eight (International Federation Federation, 2019) compounds with the strongest binding affinity than the standard drugs were decided to portray the binding mode of some autoimmune diseases and SARS-CoV-2 Mpro. The top eight (International Federation Federation, 2019) compounds are presented in Table 4. Whereas compounds (Assiri et al., 2013; Elkhalifa et al., 2018; Zhou et al., 2020; Zhou et al., 2020; Mrid et al., 2022) were found to be consistent as the first eight least binding affinities in the other receptors as presented in Table 4.

**TABLE 4:** Present and absent of the first hit compound against the selected targets.

Compound	PTPN22	T1D	T1D	RA	RA	SARS
4	√	√	√	√	√	√
10	√	√	—	—	√	—
14	√	—	—	—	—	—
16	√	√	√	√	√	√
18	√	√	√	√	√	√
23	√	√	√	√	√	√
27	√	√	—	—	—	—
30	√	—	√	√	√	√

NB: √ = Present; - = absent.

**FIGURE 5:**
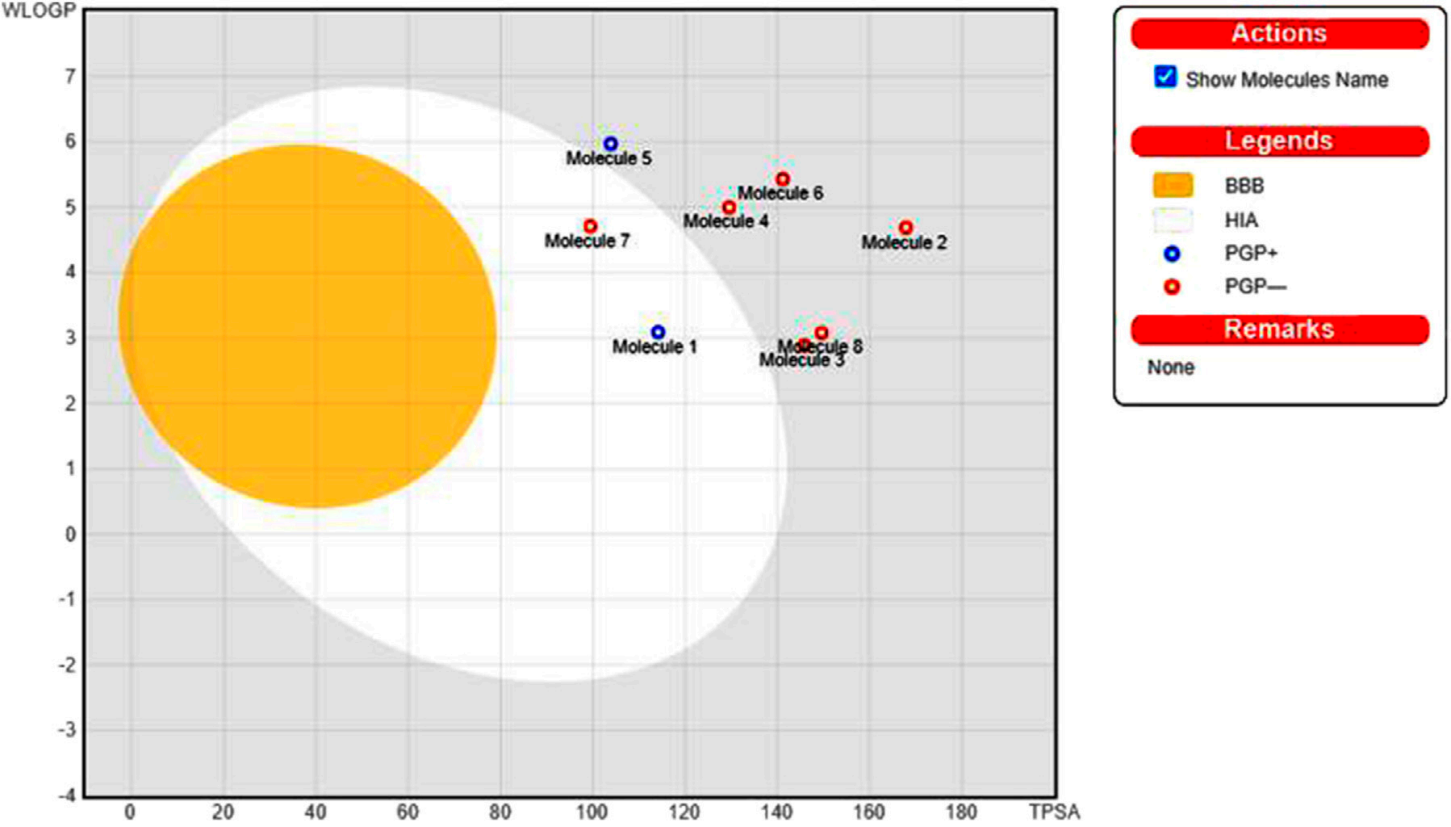
Top 8 compounds schemed on the BOILED-Egg using TPSA and WLOGP.

The pharmacological properties and toxicity of inhibitors in living beings were calculated using SwissADME (http://www.swissadme.ch/) and ADMETlab 2.0 (https://admetmesh.scbdd.com/) web servers on the top 8 compounds. According to Lipinski’s “rule of five,” (Lipinski et al., 2001) good absorption or permeation is more likely when the molecular weight (MW) is 500 Da or less, there are five or fewer hydrogen bond donors present, LogP is five, and there are ten or more hydrogen bond acceptors present. Two additional pertinent descriptors were found by Veber et al. (2002) to be the number of rotatable bonds (NBR) 10 and the polar surface area (PSA) 140 Å2. The results showed that all inhibitors satisfied Lipinski and Veber Rule. All the compounds also show moderately soluble lipid and water solubility except compounds (18 and 23) (Table 5). According to the literature, drugs typically have seven rotatable bonds, whereas toxins only have three (Khanna and Ranganathan, 2009). We discovered that the number of rotatable bonds in the compounds (Rieck et al., 2007; Elkhalifa et al., 2018; Zhou et al., 2020; Zhou et al., 2020) ranges from 0 to 7 (Table 5). The BOILED-Egg is an easy-to-use model for foretelling the biodistribution of organic compounds (Daina and Zoete, 2016). Two compounds were located inside the egg, which represents an appropriate physicochemical space for biodistribution, when we mapped WLOGP and TPSA of the virtual screening hits to the BOILED egg (Figure 5). Lead compounds found in egg white, which indicates human intestinal absorption (HIA) without blood-brain barrier (BBB) permeation, would be favored for faster drug development related to the treatment of autoimmune diseases. While 4 compounds are still close to egg white and would have better bioavailability profiles during a drug development phase, 6 compounds are in the gray area and fall into this category. Compounds inside the egg white are molecules 1 and 7, that is compounds 4 and 27, respectively.

**TABLE 5:** Calculated physicochemical properties of the top 8 compounds.

Name	Physicochemical properties					Lipid solubility		Water solubility	
	MW	Rotatable bonds	H-bond acceptors	H-bond donors	TPSA	WLOGP	Consensus log P	ESOL log S	ESOL class
Cpd 4	463.48	6	7	2	114.13	3.09	2.85	−4.75	Moderately soluble
Cpd 10	496.56	9	7	2	167.88	4.69	3.69	−5.4	Moderately soluble
Cpd 14	434.4	10	7	3	145.84	2.89	2.13	−4.29	Moderately soluble
Cpd 16	527.59	9	6	2	129.52	5	2.13	−4.29	Moderately soluble
Cpd 18	494.99	5	4	3	103.88	5.97	4.4	−6.39	Poorly soluble
Cpd 23	506.96	10	6	3	141.15	5.43	4.56	−6.33	Poorly soluble
Cpd 27	432.86	8	5	2	99.42	4.71	4.11	−5.59	Moderately soluble
Cpd 30	445.38	6	7	2	149.6	3.08	2.3	−4.51	Moderately soluble

CYP1A2, CYP2C19, CYP2C9, CYP2D6, and CYP3A4 are five major cytochrome P450 isoforms that have a significant role in drug metabolic activities and abolition. As a result, these isozymes play an important role in controlling drug reactions, which in turn can determine effectiveness and undesirable effects. Data on the ability of top virtual screening hits to inhibit significant CYP isozymes can be found in Supplementary Table S2. We discovered that compound 4, which would inhibit four isozymes, is likely to show more response to therapy. The remaining compounds may display two or three isozymes. We then calculated the toxicity statuses of the chosen compounds using the ADMETlab webserver and OSIRIS Property Explorer (Dong et al., 2018). Eight of the chosen compounds could have high toxicities, as shown in Supplementary Table S3. It should be noted that compound 4 is expected to have high reproductive effectiveness. Compound 10 is predicted to have high irritants. Compounds with high hepatotoxicity are compounds (4, 10, 16. 18, 23, and 27). Compounds with high ames mutagenicity are compounds 14 and 16. Compound 4 appears to be a better lead without significant toxicity, on the other hand.

**FIGURE 6:**
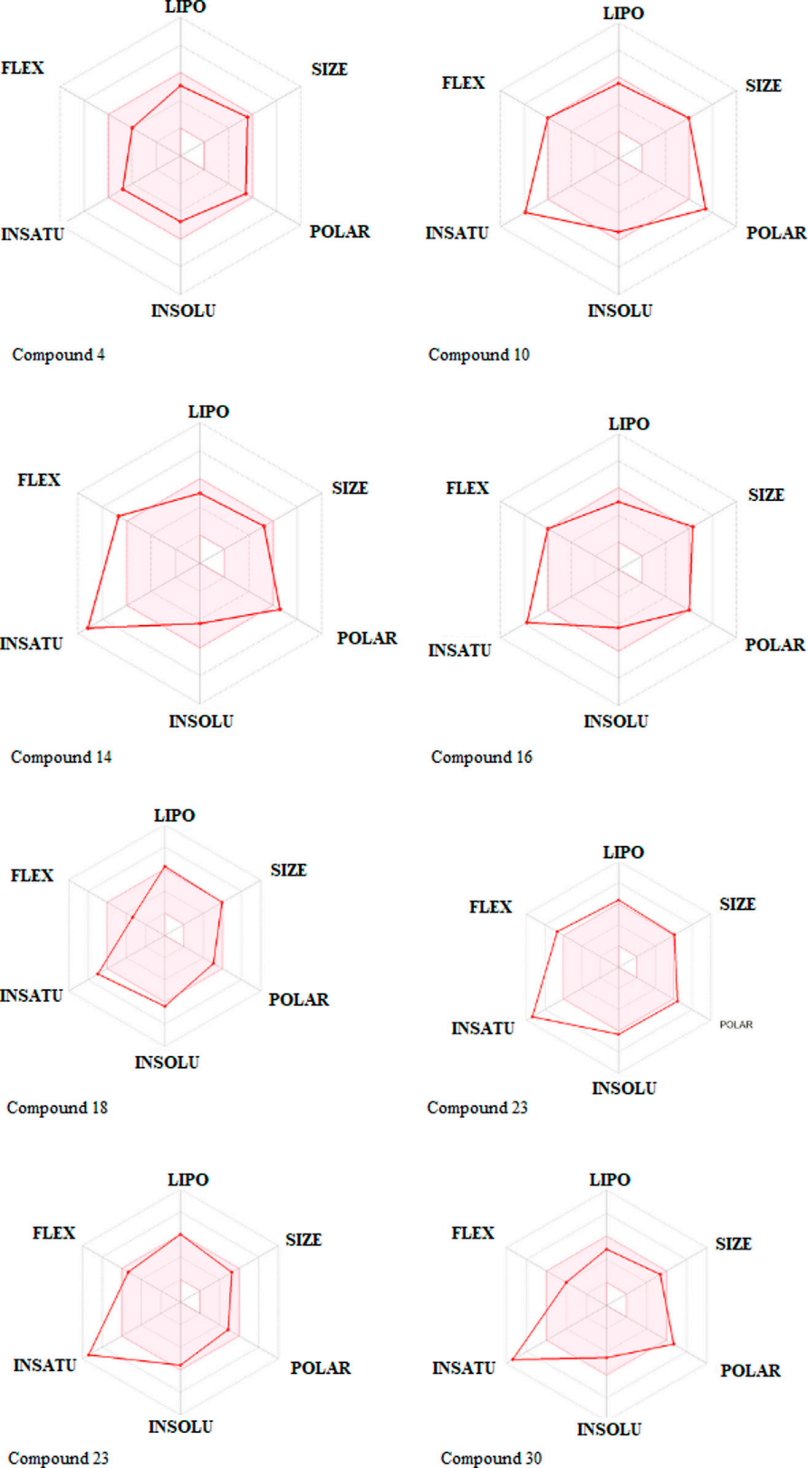
Rundown of pharmacokinetic properties of the top, restricting phytocompounds.

One compound stood out when we looked at the ADMET profiles of the top eight hits from the target identification (Figure 3). These molecules are predicted not to cross the BBB, have rheological properties suitable for absorption and bioavailability, and appear to carry no or lower toxicity risks. The radar charts show that the properties of these biomolecules that favor absorption and bioavailability are lipophilicity, size, polarity, insolubility, instauration, and flexibility (Figure 6). It’s noteworthy that com-pound 4 occupies the entire physicochemical region for absorption and bioavailability. It is also tempting to mention that, out of the top eight hits, compound 4 poses only reproductive effective as toxicity risks (Supplementary Table S3). Compound 4’s analyses, as seen in Table 3; Supplementary Table S2, revealed no offenses of these guidelines, indicating that they would exhibit well-behaved absorption or permeation.

### 3.3 Docking interactions of compound 4

**FIGURE 7:**
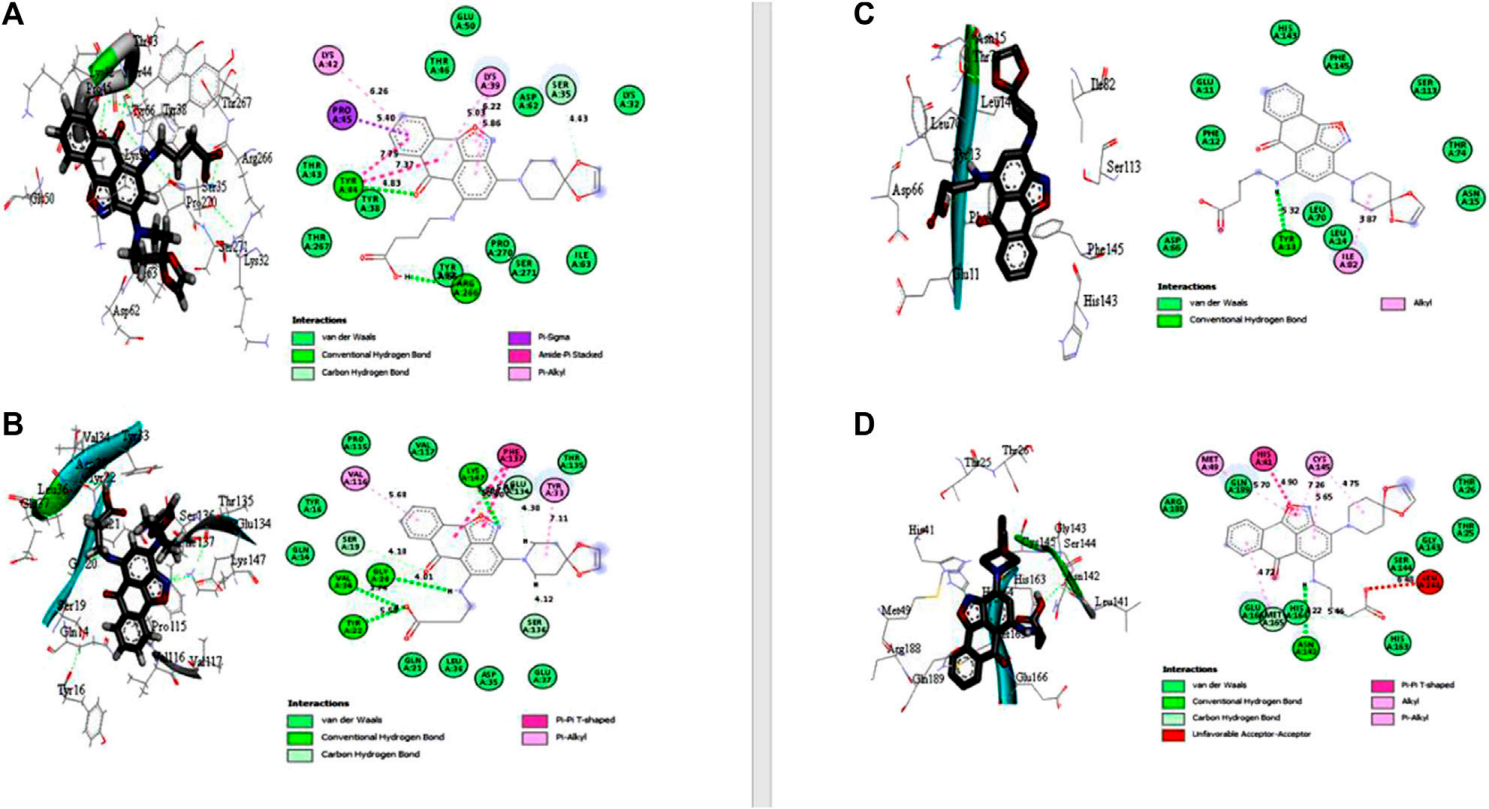
Docked poses of compound 4 with **(A)** protein tyrosine phosphatase, nonreceptor type 22 (lymphoid) isoform 1 (PTPN22) (PDB ID: 4J51), **(B)** Type 1 diabetes (PDB ID: 1JK8), **(C)** Rheumatoid arthritis (PDB ID: 2FSE), and **(D)** SARS-CoV- 2 (PDB ID: 6LU7).

Under the results of toxicity and ADME analyses of the top eight compounds, compound 4 with favored oral bioavailability attributes was selected for docking visualization/interactions with the receptors. Figure 7 displays numerous interactions of compound 4 with the least docking score with PTPN22 (4J51.pdb), diabetes type 1 (1JK8.pdb), and rheumatoid arthritis (2FSE.pdb) also interact with the critical residues of the SARS-CoV-2 main protease (6LU7.pdb). Molecular docking calculations confirmed that compound 4 can occupy the catalytic sites of all the receptors and produce a net of hydrogen (Figures 7A–D). From the results, it has been observed that compound 4 formed two conventional hydrogen bonds and one carbon-hydrogen bonds interactions with the PTPN22 receptor was shown in Figure 7A. Docking analysis of the PTPN22 receptor with compound 4 allowed us to recognize certain residues, viz. ARG266, TYR44, PRO45, LYS42, LYS39, and SER35, within the PTPN22 receptor binding pocket, which is crucial to ligand binding affinity. The modes of interaction between compound 4 and the active site diabetes type 1 (PDB ID: 1JK8) are shown in Figure 7B, which shows four conventional hydrogen bonds and three carbon-hydrogen bond interactions between the ligand and amino acids GLY20, VAL34, TYR22, LYS147, SER19, GLU134, and SER136 respectively. Thus, the one pi-pi T-shaped bonds with the amino acids PHE137. In addition, two pi-alkyl-type interactions are observed between the compound and the amino acids VAL116 and TYR33, respectively. However, compound 4 does not show any carbon-hydrogen-like bond with rheumatoid arthritis (PDB ID: 2FSE). The two-dimensional visualization indicates that compound 4 inter-acted with the amino acid TYR13 via conventional hydrogen bonds. Compound 4 was also bound to the ILE82 site by an alkyl bond. Additional van der Waals bonds also occurred at the ASP66, PHE12, GLU11, HIS143, PHE154, SER113, THR74, ASN15, LEU70, and LEU14 sites (Figure 7C). These results provide evidence of the critical role of the amino acids tyrosine (TYR), lysine (LYS), and serine (SER) for the stability of the compound 4 in the active site of autoimmune receptors. Therefore, these amino acid residues should be taken into account to improve the biological inhibitory activity of compound 4 analogs against autoimmune disorders.

The two crucial peptides in the catalytic site of the SARS-CoV-2 target site are CYS145 and HIS41. Studies have shown that the catalytic dyad formed by CYS145 and HIS41 increases the reactivity of the nucleophile by acting as a base and a nucleophile, respectively. The SARS-CoV-2 Mpro is then released after the inhibitor attacks the dyad to create an alternate complex. Compound 4 interacts with CYS145 and HIS41 in our study, indicating that these substances may have inhibitory activity against SARS-coronavirus-2 Mpro. Asymmetric aromatic disulfides may fight with the substrate in the SARS-CoV-2 Mpro cysteine protease as an inhibitor (competitive inhibitor). We think compound 4 has the potential to be an enzyme inhibitor and, as a result, could be a drug candidate for both autoimmune disease and the SARS-CoV-2 virus. An *in vitro* study against autoimmune disorder and SARS-CoV-2 Mpro is advised to verify this hypothesis.

### 3.4 Comparing the docking poses of compound 4 and the standard drugs

In the case of the PTPN22 enzyme, the control compound (Sulfasalazine) has the highest docking affinity than the rest of the standard drugs. The sulfasalazine-4J51 complex was stabilized by the formation of conventional hydrogen bonds with SER35 (4.22 Å), SER271 (4.17 Å), and THR46 (3.59 Å) of protein with PTPN22. The complex was also stabilized by hydrophobic interactions, including pi-pi T-shaped interaction with TYR44, Amide-pi stacked bond with TYR66, and pi-alkyl bonds (with PRO270, PRO45, LYS39, and LYS42), as shown in Supplementary Figure S1A. The amino acid residue for compound 4 and sulfasalazine complexes revealed from docking simulations results shows that (GLY20, LYS147, VAL34, TYR22, SER35, SER271, and THR46) are essential for the binding of ligands to the enzyme of PTPN22.

Furthermore, for the type 1 diabetes receptor, among the two control drugs, sotagliflozin has the highest docked score of −7.3 kcal/mol. Sotagliflozin has no single hydrogen bond but was stabilized with the formation of hydrophobic interactions, including Alkyl interactions with TYR33, VAL116, TYR16, pi-Alkyl interaction with LEU36, and pi-pi- T-shaped interaction with PHE137. The most common amino acid residue involved in the interactions of compound 4 and sotagliflozin drugs is TYR33, LEU36, PHE137, VAL116, and TYR16. The docking interactions of both compounds revealed that GLY20, VAL34, TYR55, SER19, TYR33, LEU36, PHE137, VAL116, and TYR16 are essential for the binding of ligand to the enzyme of type 1 diabetes (PDB id: 1JK8).

Here, for rheumatoid arthritis protein, sulfasalazine (standard drug) has the highest docked score of −7.7 kcal/mol. In the complex of PDB id 2FSE with sulfasalazine, three conventional Hydrogen bonds were formed by sulfasalazine with ARG44 (5.08 Å), TYR150 (6.92 Å), and ASP29 (3.55 Å) of 2FSE (Supplementary Figure S1C). Other than conventional H-bonds, the hydrophobic interactions such as pi-Alkyl bonds (with LEU45, ILE31, ALA52), pi-pi stacked bonds (with PHE28), pi-pi T-shaped bonds (with PHE28), and electrostatic interaction (pi-cation) with ARG44 were involved in the stabilization of the complex. The amino acid residue for compound 4 and sulfasalazine complexes against rheumatoid arthritis revealed from docking simulations results shows that TYR13, ARG44, TYR150, ASP29, and ARG44 are essential for the binding of ligands to the enzyme (PDB id 2FSE).

Lastly, for the SARS-CoV-2 protein (PDB id: 6LU7), the control drug hydroxychloroquine has a binding affinity of −6.3 kcal/mol. Hydroxychloroquine formed two conventional hydrogen bonds with THR190 (4.22 and 4.38 Å) of SARS-CoV-2 protein in the hydroxychloroquine-6LU7 complex (Supplementary Figure S1D). Further, this complex was also stabilized by one carbon-hydrogen bond with GLN189 (4.43 Å), one pi-cation ionic interaction with HIS41, and hydrophobic (pi-Alkyl and Alkyl) interactions with CYS145 and MET49. The common amino acids involved in the interactions with compound 4 and the standard drug hydroxychloroquine are HIS41, CYS145, and MET49. Therefore, the interactions revealed that amino acids ASN142, MET165, THR190, GLN189, HIS41, CYS145, and MET49 are essential for the binding of ligands to the enzyme (PDB id 6LU7). Dhankhar et al. (2020), Kumar et al. (2021), and Dhankhar et al. (2021), examined research on ligand access channels in SARS-CoV-2 receptor and found that amino acid residues Met49, Cys145, and Gln189 in particular may play a significant function in ligand binding interactions. Our data corroborate that hypothesis and identify Met49, Cys145, and Gln189 as an amino acid residues implicated in the binding of SARS-CoV-2 Mpro.

### 3.5 Molecular dynamics (MD) simulations analysis

**FIGURE 8:**
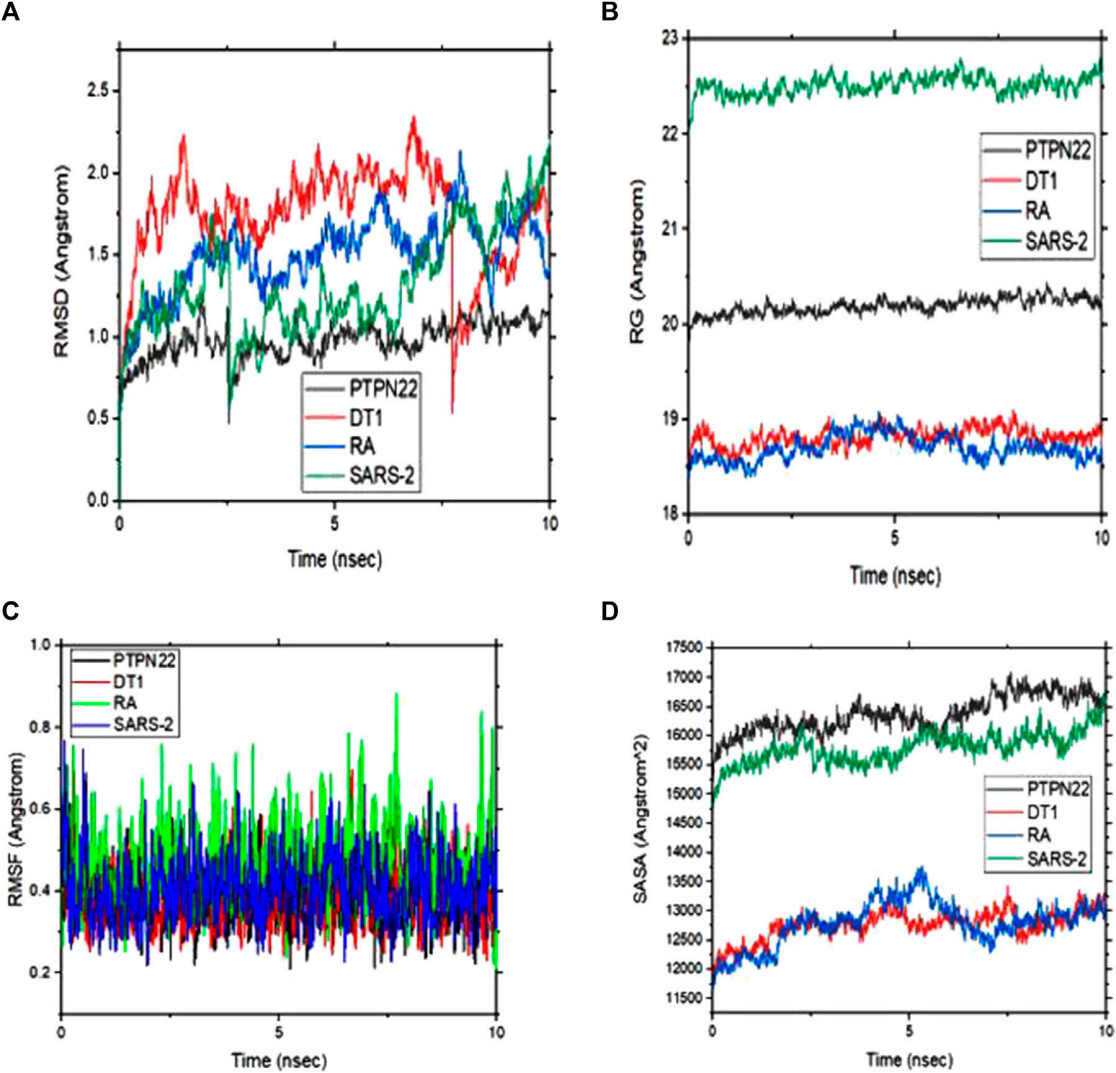
**(A)** Root means square deviation (RMSD) plots of backbone atoms of the four selected complexes **(B)** Radius of gyration (RG) of the four selected complexes **(C)** Root mean square fluctuation (RMSF) plots of backbone atoms of the four selected complexes and **(D)** solvent accessible surface area (SASA) of the four complexes during 10 ns.

The energetic motion and level of stability of the complexes were examined and understood using the MD simulation. MD simulation for all the complexes of compound 4 is performed for 10 ns (Figure 8). RMSD of alpha carbon atoms, the radius of gyration (RG), RMSF, and solvent accessible surface area (SASA) of the complexes are investigated. Figure 8A shows how compound 4 complexes are stable before 10 ns and how the RMSD of the protein backbone and compound 4 are related. Interestingly, none of the complexes showed RMSD values greater than 2, which supports the strict specificity of the most active complexes. Throughout the remainder of the simulation, these complexes kept their RMSD profile stable. When compound 4 is tested against the PTPN22 protein, it exhibits the lowest RMSD value compared with the remaining complexes, indicating that it is more stable and stays in the protein’s pouch (Table 6). To determine the stability of receptors through MD simulation, the RG of complexes with compound 4 was also estimated (Figure 8B). In comparison to other receptors, the rheumatoid arthritis receptor generally exhibits less variation in RG values. The average RG values of receptors with compound 4 are 20.19, 18.82, 18.70, and 22.52 Å, respectively. The complexes’ stability is indicated by this small value (Table 6). The RMSF study provided insightful data on the structural fluctuations of various protein regions. Increased variations in the residues indicate that the protein is less stable. The consistency of compound 4 with the sample protein is indicated by the fact that the RMSF of the complexes, which is depicted in Figure 7C, remained below 0.5 for most of the receptors’ amino acids. Nevertheless, a few variations are seen at the terminal, which might be a result of these residues’ high plank position in the rheumatoid arthritis receptor. The SARS-CoV-2 Mpro receptor exhibits some variations as well, which could be brought on by compound 4’s dynamic characteristics in the bonding zone. To further examine the trajectories of compound 4, the average RMSF of com-pound 4 was also calculated. This calculation revealed some variation, indicating a kinetic shift from their start point. Compound 4 must therefore be categorized as a drug. Additionally, following MD simulation, the lowest RMSF value of compound 4 against type 1 diabetes (PDB ID: 1JK8) was also discovered. Calculating the changes in SASA allowed for further confirmation of the stability. The SASA of the receptors with compound 4 are shown in Figure 8D. The SASA of diabetes type 1 with compound 4 complex is similar to rheumatoid arthritis with compound 4. Likewise, PTPN22 and SARS-CoV-2 with compound 4 are similar, further confirming the stability of compound 4 with all the protein crystal structures. The average SASA values for all the receptors-compound 4 are presented in Table 6.

**TABLE 6:** Calculated free binding energy for the complexes estimated using MM/GBSA analysis and the values of the average variation.

Parameter	PTPN22	DT1	RA	SARS-2
δ E (internal)	X	10.5977	12.7555	−19.6068
δ E (electrostatic) + δ G (sol)	X	−17.4634	−29.3878	−5.8592
δ E (VDW)	X	−38.2881	−25.4669	−38.1391
δ G binding (kcal/mol)	X	−45.1538±0.3823	−42.0993±0.4699	−63.6051 ± 0.464
RMSD (Å)	0.98	1.74	1.50	1.33
RG (Å)	20.19	18.82	18.70	22.52
RMSF (Å)	0.39	0.38	0.47	0.41
SASA ( ${Å}^{2}$ )	16,368.53	12,768.89	12,794.09	15,792.73

**FIGURE 9:**
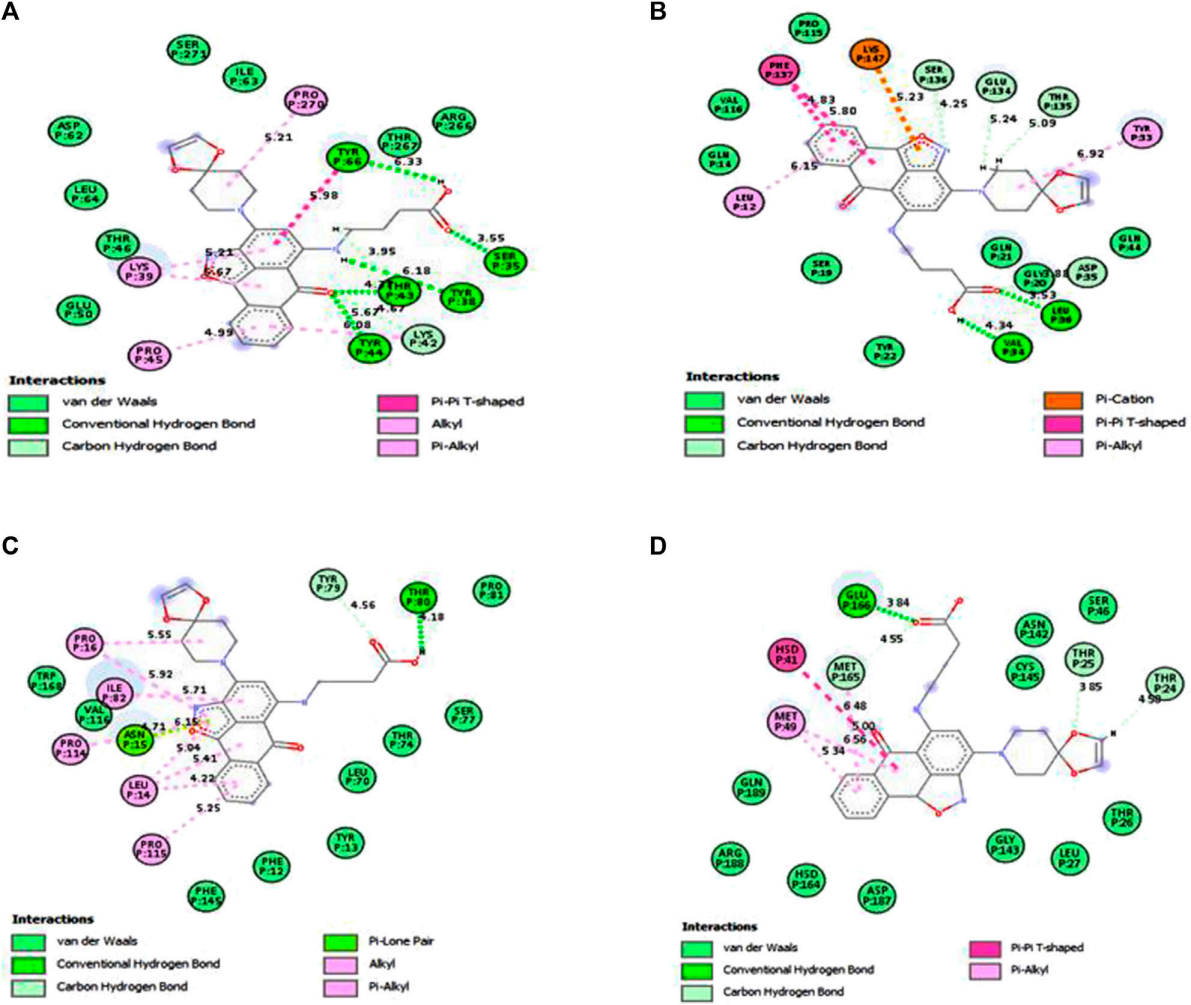
Interactions of compound 4 and key residues for **(A)** PTPN22, **(B)** type 1 diabetes, **(C)** rheumatoid arthritis, and **(D)** SARS-CoV-2 main protease receptors after simulations.

The MM/GBSA approach is used to evaluate the calculated free binding energy of compound 4 from each receptor. The free binding energy for each complex was calculated using the molecular dynamics trajectory from the previous 2 ns (100) frames. The effects of other non-bonded interaction energies were predicted along with the calculated free binding energy of each compound 4-receptor complex. With diabetes type 1 protein, compound 4 has a calculated free binding energy of −45.1538 ± 0.3823 kcal/mol, whereas, with rheumatoid arthritis protein, compound 4 has a calculated binding energy of −42.0993 ± 0.4699 kcal/mol. Interactions like internal energy, van der Waals energy, electrostatic, and solvation energy is governed. Across all types of interactions, the van der Waals and the sum of electrostatic and the calculated free binding energy were mainly influenced by solvation energies. The internal energy, on the other hand, made the smallest contribution to the final calculated free binding energies. Moreover, the internal energy interaction values of compound 4 and SARS-CoV-2 Mpro protein complexes demonstrated high contribution after van der Waals energy (Table 6). Figure 9A shows that compound 4 in the catalytic pocket of the PTPN22 protein has undergone a significant geometric change in the pose after simulation (10 ns), and Figures 9B–D shows that compound 4 in the catalytic pocket of DT1 and RA has also undergone a significant elongated change after the simulation (10 ns) (curved to straight). Effectively receptor—ligand acquisition and interactions with residues result from these conformational changes, which increase stability and binding energy (shown in Table 6). Therefore, MM-GBSA calculations from MD simulation trajectories were well supported by the binding affinity found in the docking results. In addition, the last frame (10 ns) of MD simulation showed the positional change of the compound 4 and protein targets as compared to docking (shown in Figure 7), indicating the best binding position for effectively fitting in the catalytic pocket of the protein (see Figure 9).

To further analyze the binding between compound 4 and the receptor complexes, the contactFreq.tcl module in VMD (cut-off of 4 Å) was used. The proportion of compound 4 contact frequency with the receptors’ binding amino acid residues was reported, and the results indicate that certain residues are engaged in compound 4’s ongoing interactions with those residues (Supplementary Table S4).

## 4 Conclusion

Several 3D QSAR methods are used in the current article to explore the design constraints of anti-autoimmune disease inhibitors. The most suitable model was chosen based on its higher levels of internal and external predictability. We have employed standard statistical parameters to assess the efficacy of these methods. In general, it can be concluded that UVEPLS and FFDSEL CoMFA analyses produced similar findings in terms of the compounds’ structural requirements. Molecular docking was performed on 31 compounds known as an anti-autoimmune disorder. A large number of these compounds have demonstrated impressive binding interactions with SARS-coronavirus-2 Mpro and autoimmune receptors. The results have demonstrated that the chosen compound 4, in-silicoly, has a strong ability to combat SARS-coronavirus-2 Mpro and autoimmune receptors. The location and perception of compound 4 in the catalytic site changed after the MD simulation was complete. This significant finding demonstrated the value of using MD simulation after docking compound 4. The ligand-receptor complex from the molecular dynamics simulation revealed order-binding residues, changed the other residues in the catalytic site identified by docking, and revealed some new residues that were nearby compound 4 and might be involved in the interaction. To determine whether compound 4 could be a drug applicant to cure autoimmune diseases and SARS-coronavirus-2, additional *in vitro* and *in vivo* research needs to be done. This research may represent an *in silico* strategy for the discovery of brand-new inhibitors of anti-autoimmune disorders and anti-SARS-coronavirus-2.

## Data Availability

The original contributions presented in the study are included in the article/Supplementary Material, further inquiries can be directed to the corresponding authors.
