# Supplementary material for: Combining docking, molecular dynamics simulations, AD-MET pharmacokinetics properties, and MMGBSA calculations to create specialized protocols for running effective virtual screening campaigns on the autoimmune disorder and SARS-CoV-2 main protease
Source: Front Mol Biosci. 2023 Sep 1;10:1254230. doi: 10.3389/fmolb.2023.1254230 (PMC10523577; doi:10.3389/fmolb.2023.1254230)
Supplement: Supplementary file 1 [file Table1.docx]

**Supplementary material**

Table S1. Molecular docking results for all the 31 compounds and the reference drugs.

| s/n | PTPN22 (PDB ID: 4J51) | | | DT1 (PDB ID: 1JK8) | | | DT1 (PDB ID: 1XW7) | | | RA (PDB ID: 2AXJ) | | | RA (PDB ID: 2FSE) | | | SC2 (PDB ID: 6LU7) | |
| --- | --- | --- | --- | --- | --- | --- | --- | --- | --- | --- | --- | --- | --- | --- | --- | --- | --- |
|  | Affinity | Est. Ki | LE | Affinity | Est. Ki | LE | Affinity | Est. Ki | LE | Affinity | Est. Ki | LE | Affinity | Est. Ki | LE | Affinity | Est. Ki |
| 1 | -8.1 | 1.16 uM | -0.39 | -7.0 | 7.40 uM | -0.33 | -5.7 | 66.36 uM | -0.27 | -6.0 | 39.99 uM | -0.29 | -6.9 | 8.76 uM | -0.33 | -7.4 | 3.77 uM |
| 2 | -7.9 | 1.62 uM | -0.46 | -6.6 | 14.53 uM | -0.39 | -6.0 | 39.99 uM | -0.35 | -6.2 | 28.53 uM | -0.36 | -6.4 | 20.36 uM | -0.38 | -6.7 | 12.27 uM |
| 3 | -7.5 | 3.18 uM | -0.38 | -6.8 | 10.37 uM | -0.34 | -5.0 | 0.22 mM | -0.25 | -5.4 | 0.11 mM | -0.27 | -6.0 | 39.99 uM | -0.30 | -6.8 | 10.37 uM |
| 4 | -9.7 | 77.60 nM | -0.29 | -8.6 | 496.78 nM | -0.25 | -6.6 | 14.53 uM | -0.19 | -7.4 | 3.77 uM | -0.22 | -8.5 | 588.12 nM | -0.25 | -8.6 | 496.78 nM |
| 5 | -6.4 | 20.36 uM | -0.46 | -5.4 | 0.11 mM | -0.39 | -4.2 | 0.83 mM | -0.30 | -4.5 | 0.50 mM | -0.32 | -5.1 | 0.18 mM | -0.36 | -5.6 | 78.56 uM |
| 6 | -7.0 | 7.40 uM | -0.47 | -5.6 | 78.56 uM | -0.37 | -4.5 | 0.50 mM | -0.30 | -4.9 | 0.26 mM | -0.33 | -5.3 | 0.13 mM | -0.35 | -6.1 | 33.78 uM |
| 7 | -6.2 | 28.53 uM | -0.33 | -5.2 | 0.15 mM | -0.27 | -4.4 | 0.60 mM | -0.23 | -4.7 | 0.36 mM | -0.25 | -4.7 | 0.36 mM | -0.25 | -6.0 | 39.99 uM |
| 8 | -8.6 | 496.78 nM | -0.31 | -7.8 | 1.92 uM | -0.28 | -6.1 | 33.78 uM | -0.22 | -6.8 | 10.37 uM | -0.24 | -7.5 | 3.18 | -0.27 | -7.4 | 3.77 uM |
| 9 | -8.9 | 299.41 nM | -0.30 | -8.5 | 588.12 nM | -0.28 | -6.4 | 20.36 uM | -0.21 | -7.0 | 7.40 uM | -0.23 | -7.8 | 1.92 uM | -0.26 | -8.2 | 975.81 nM |
| 10 | -9.1 | 213.63 nM | -0.27 | -8.7 | 419.63 nM | -0.26 | -6.3 | 24.10 uM | -0.19 | -6.8 | 10.37 uM | -0.20 | -8.7 | 419.63 nM | -0.26 | -7.6 | 2.69 uM |
| 11 | -8.5 | 588.12 nM | -0.33 | -7.4 | 3.77 uM | -0.28 | -6.7 | 12.27 uM | -0.26 | -6.4 | 20.36 uM | -0.25 | -7.3 | 4.46 uM | -0.28 | -7.5 | 3.18 uM |
| 12 | -8.8 | 354.46 nM | -0.26 | -8.0 | 1.37 uM | -0.24 | -6.3 | 24.10 uM | -0.19 | -7.0 | 7.40 uM | -0.21 | -7.8 | 1.92 uM | -0.23 | -8.1 | 1.16 uM |
| 13 | -8.5 | 588.12 nM | -0.29 | -7.9 | 1.62 uM | -0.27 | -6.5 | 17.20 uM | -0.22 | -6.9 | 8.76 uM | -0.24 | -7.5 | 3.18 uM | -0.26 | -8.1 | 1.16 uM |
| 14 | -9.0 | 252.91 nM | -0.28 | -7.9 | 1.62 uM | -0.25 | -6.0 | -6.0 uM | -0.19 | -6.6 | 14.53 uM | -0.21 | -8.1 | 1.16 uM | -0.25 | -7.9 | 1.62 uM |
| 15 | -8.9 | 299.41 nM | -0.23 | -8.2 | 975.81 nM | -0.22 | -7.4 | 3.77 uM | -0.19 | -6.5 | 17.20 uM | -0.17 | -8.2 | 975.81 nM | -0.22 | -8.2 | 975.81 nM |
| 16 | -9.0 | 252.91 nM | -0.24 | -9.4 | 128.75 nM | -0.25 | -7.0 | 7.40 uM | -0.18 | -7.4 | 3.77 uM | -0.19 | -9.0 | 252.91 nM | -0.24 | -9.1 | 213.63 nM |
| 17 | -8.2 | 975.81 nM | -0.22 | -8.1 | 1.16 uM | -0.22 | -6.1 | 33.78 uM | -0.16 | -6.8 | 10.37 uM | -0.18 | -7.4 | 3.77 uM | -0.20 | -8.0 | 1.37 uM |
| 18 | -9.6 | 91.87 nM | -0.28 | -9.2 | 180.45 nM | -0.27 | -7.1 | 6.25 uM | -0.21 | -7.8 | 1.92 uM | -0.23 | -9.2 | 180.45 nM | -0.27 | -8.3 | 824.26 nM |
| 19 | -8.3 | 824.26 nM | -0.29 | -8.0 | 1.37 uM | -0.28 | -6.6 | 14.53 uM | -0.23 | -7.1 | 6.25 uM | -0.24 | -8.1 | 1.16 uM | -0.28 | -7.9 | 1.62 uM |
| 20 | -8.4 | 696.25 nM | -0.37 | -7.3 | 4.46 uM | -0.32 | -6.2 | 28.53 uM | -0.27 | -6.4 | 20.36 uM | -0.28 | -7.6 | 2.69 uM | -0.33 | -7.7 | 2.27 uM |
| 21 | -7.2 | 5.28 uM | -0.42 | -6.0 | 39.99 uM | -0.35 | -5.1 | 0.18 mM | -0.30 | -4.9 | 0.26 mM | -0.29 | -5.8 | 56.05 uM | -0.34 | -6.2 | 28.53 uM |
| 22 | -8.9 | 299.41 nM | -0.33 | -8.1 | 1.16 uM | -0.30 | -6.6 | 14.53 uM | -0.24 | -7.2 | 5.28 uM | -0.27 | -7.5 | 3.18 uM | -0.28 | -7.6 | 2.69 uM |
| 23 | -9.0 | 252.91 nM | -0.26 | -8.5 | 588.12 nM | -0.24 | -7.5 | 3.18 uM | -0.21 | -7.7 | 2.27 uM | -0.22 | -8.4 | 696.25 nM | -0.24 | -8.3 | 824.26 nM |
| 24 | -8.2 | 975.81 nM | -0.26 | -8.4 | 696.25 nM | -0.27 | -6.3 | 24.10 uM | -0.20 | -7.5 | 3.18 uM | -0.24 | -7.8 | 1.92 uM | -0.25 | -7.8 | 1.92 uM |
| 25 | -8.8 | 354.46 nM | -0.30 | -8.4 | 696.25 nM | -0.29 | -6.0 | 39.99 uM | -0.21 | -7.2 | 5.28 uM | -0.25 | -8.1 | 1.16 uM | -0.28 | -8.2 | 975.81 nM |
| 26 | -8.1 | 1.16 uM | -0.25 | -7.8 | 1.92 uM | -0.24 | -6.4 | 20.36 uM | -0.20 | -6.6 | 14.53 uM | -0.21 | -7.5 | 3.18 uM | -0.23 | -7.7 | 2.27 uM |
| 27 | -9.1 | 213.63 nM | -0.29 | -8.7 | 419.63 nM | -0.28 | -6.5 | 17.20 uM | -0.21 | -6.9 | 8.76 uM | -0.22 | -7.8 | 1.92 uM | -0.25 | -8.2 | 975.81 nM |
| 28 | -8.4 | 696.25 nM | -0.32 | -7.6 | 2.69 uM | -0.29 | -6.2 | 28.53 uM | -0.24 | -6.6 | 14.53 uM | -0.25 | -7.6 | 2.69 uM | -0.29 | -7.3 | 4.46 uM |
| 29 | -8.8 | 354.46 nM | -0.26 | -8.5 | 588.12 nM | -0.25 | -6.5 | 17.20 uM | -0.19 | -7.0 | 7.40 uM | -0.21 | -8.2 | 975.81 nM | -0.24 | -7.6 | 2.69 uM |
| 30 | -10.2 | 33.37 nM | -0.31 | -8.2 | 975.81 nM | -0.25 | -6.7 | 12.27 uM | -0.20 | -7.3 | 4.46 uM | -0.22 | -8.6 | 496.78 nM | -0.26 | -9.1 | 213.63 nM |
| 31 | -8.6 | 496.78 nM | -0.25 | -8.2 | 975.81 nM | -0.24 | -6.4 | 20.36 uM | -0.19 | -7.0 | 7.40 uM | -0.21 | -7.7 | 2.27 uM | -0.23 | -8.4 | 696.25 |
| ST1 | -8.1 | 1.16 uM | -0.34 |  |  |  |  |  |  | -5.7 | 66.36 uM | -0.24 | -7.5 | 3.18 uM | -0.31 |  |  |
| ST2 | -7.8 | 1.92 uM | -0.41 |  |  |  |  |  |  | -5.7 | 66.36 uM | -0.30 | -7.0 | 7.40 uM | -0.37 |  |  |
| ST3 | -8.1 | 1.16 uM | -0.35 |  |  |  |  |  |  | -6.1 | 33.78 uM | -0.27 | -6.6 | 14.53 uM | -0.29 |  |  |
| ST4 |  |  |  | -4.3 | 0.70 mM | -0.48 | -3.6 | 2.30 mM | -0.40 |  |  |  |  |  |  |  |  |
| ST5 | -8.7 | 419.63 nM | -0.31 |  |  |  |  |  |  | -7.2 | 5.28 uM | -0.26 | -7.7 | 2.27 uM | -0.28 |  |  |
| ST6 | -7.0 | 7.40 uM | -0.30 |  |  |  |  |  |  | -5.4 | 0.11 mM | -0.23 | -6.0 | 39.99 uM | -0.26 | -6.3 | 24.10 uM |
| ST7 |  |  |  | -7.3 | 4.46 mM | -0.26 | -5.8 | 56.05 uM | -0.21 |  |  |  |  |  |  |  |  |

NB: ST1 = Etoricoxib; ST2 = Leflunomide; ST3 = Meloxicam; ST4 = Metformin; ST5 =sulfasalazine; ST6 = Hydroxychloroquine; ST7 = Sotagliflozin

Table S2. Calculated metabolic, elimination profiles, number of violations, pains alerts, and synthetic accessibility of the selected compounds.

| Cpd | GI absorption | BBB permeant | Pgp substrate | CYP1A2 inhibitor | CYP2C19 inhibitor | CYP2C9 inhibitor | CYP2D6 inhibitor | CYP3A4 inhibitor | log Kp (cm/s) | Lipinski violations | Veber violations | Bioavailability Score | PAINS alerts | Leadlikeness violations | Synthetic Accessibility |
| --- | --- | --- | --- | --- | --- | --- | --- | --- | --- | --- | --- | --- | --- | --- | --- |
| 4 | High | No | Yes | No | Yes | Yes | Yes | Yes | -6.76 | 0 | 0 | 0.56 | 2 | 1 | 4.6 |
| 10 | Low | No | No | No | Yes | Yes | No | Yes | -6.38 | 0 | 1 | 0.55 | 0 | 3 | 4.04 |
| 14 | Low | No | No | No | Yes | Yes | No | No | -6.69 | 0 | 1 | 0.55 | 0 | 2 | 3.2 |
| 16 | Low | No | No | No | Yes | Yes | No | No | -6.69 | 0 | 1 | 0.55 | 0 | 2 | 3.2 |
| 18 | Low | No | Yes | Yes | No | Yes | No | Yes | -5.47 | 0 | 0 | 0.56 | 1 | 2 | 4.47 |
| 23 | Low | No | No | No | Yes | Yes | No | Yes | -5.42 | 1 | 1 | 0.56 | 0 | 3 | 3.53 |
| 27 | High | No | No | No | Yes | Yes | No | Yes | -5.37 | 0 | 0 | 0.56 | 0 | 3 | 2.99 |
| 30 | Low | No | No | No | Yes | Yes | No | Yes | -6.88 | 0 | 1 | 0.56 | 0 | 1 | 2.85 |

Table S3. Toxicity profiles of the selected compounds.

| Compound Number | ADMETlab | | | OSIRIS | | | | |
| --- | --- | --- | --- | --- | --- | --- | --- | --- |
|  | hERG blocker | Hepatotoxicity | Ames mutagenicity | Drug-likeness | Mutagenic | Tumorigenic | Reproductive Effective | Irritant |
| 4 | No | High | No | -0.981 | none | none | high | none |
| 10 | No | High | No | 2.758 | none | low | low | high |
| 14 | Medium | No | High | -0.439 | none | none | none | none |
| 16 | No | High | High | 5.363 | none | none | none | low |
| 18 | No | High | No | -3.236 | none | none | none | none |
| 23 | No | High | No | 5.746 | none | none | none | none |
| 27 | High | High | No | -1.927 | none | none | none | none |
| 30 | No | Low | No | -3.260 | none | none | none | none |

Table S4. Contact frequency (%) analysis of compound 4-receptors complexes after 10 ns MD simulations.

| PTPN22: Compound4-4J51 | | Type 1 diabetes: compound4-1Jk8 | | Rheumatoid arthritis Compound4-2AXJ | | SARS-CoV-2 compound4-6lu7 | |
| --- | --- | --- | --- | --- | --- | --- | --- |
| Find interactions: | | Find interactions: | | Find interactions: | | Find interactions: | |
| Residue | fraction (%) | Residue | fraction (%) | Residue | fraction (%) | Residue | fraction (%) |
| PROA-GLN-34 | 0.2977 | PROA-LEU-12 | 0.9254 | PROA-GLU-11 | 0.033 | PROA-THR-24 | 0.9973 |
| PROA-SER-35 | 100 | PROA-GLN-14 | 0.0219 | PROA-PHE-12 | 0.487 | PROA-THR-25 | 0.996 |
| PROA-TYR-38 | 0.9813 | PROA-SER-19 | 0.4912 | PROA-TYR-13 | 0.51 | PROA-THR-26 | 100 |
| PROA-LYS-39 | 100 | PROA-GLY-20 | 100 | PROA-LEU-14 | 0.878 | PROA-LEU-27 | 0.3029 |
| PROA-LYS-42 | 0.996 | PROA-GLN-21 | 100 | PROA-ASN-15 | 0.954 | PROA-HSD-41 | 100 |
| PROA-THR-43 | 100 | PROA-TYR-22 | 0.4737 | PROA-PRO-16 | 0.435 | PROA-CYS-44 | 0.0456 |
| PROA-TYR-44 | 100 | PROA-GLU-30 | 0.0132 | PROA-ASP-66 | 0.018 | PROA-SER-46 | 0.1743 |
| PROA-PRO-45 | 0.8879 | PROA-TYR-33 | 100 | PROA-ASN-69 | 0.005 | PROA-ASP-48 | 0.0013 |
| PROA-THR-46 | 0.9413 | PROA-VAL-34 | 100 | PROA-LEU-70 | 0.782 | PROA-MET-49 | 100 |
| PROA-GLU-50 | 0.0053 | PROA-ASP-35 | 0.9868 | PROA-MET-73 | 0.608 | PROA-LEU-50 | 0.0013 |
| PROA-ASP-62 | 0.7837 | PROA-LEU-36 | 0.9956 | PROA-THR-74 | 0.655 | PROA-PRO-52 | 0.0818 |
| PROA-ILE-63 | 0.1015 | PROA-GLU-37 | 0.0132 | PROA-SER-77 | 0.787 | PROA-TYR-54 | 0.0925 |
| PROA-LEU-64 | 0.0961 | PROA-GLN-44 | 0.8114 | PROA-TYR-79 | 0.171 | PROA-ASN-119 | 0.0523 |
| PROA-TYR-66 | 100 | PROA-PRO-115 | 0.8596 | PROA-THR-80 | 0.459 | PROA-LEU-141 | 0.0067 |
| PROA-ARG-266 | 0.7156 | PROA-VAL-116 | 0.1711 | PROA-PRO-81 | 0.271 | PROA-ASN-142 | 0.7373 |
| PROA-THR-267 | 0.8798 | PROA-GLU-134 | 0.9123 | PROA-ILE-82 | 0.946 | PROA-GLY-143 | 0.5268 |
| PROA-PRO-270 | 0.5955 | PROA-THR-135 | 0.9956 | PROA-SER-113 | 0.021 | PROA-SER-144 | 0.059 |
| PROA-SER-271 | 0.2043 | PROA-SER-136 | 0.9605 | PROA-PRO-114 | 0.631 | PROA-CYS-145 | 0.9987 |
|  | | PROA-PHE-137 | 100 | PROA-PRO-115 | 0.784 | PROA-HSD-163 | 0.1971 |
|  |  | PROA-LYS-147 | 0.8947 | PROA-VAL-116 | 0.117 | PROA-HSD-164 | 0.9196 |
|  |  |  | | PROA-HSD-143 | 0.008 | PROA-MET-165 | 0.9987 |
|  |  |  |  | PROA-PHE-145 | 0.424 | PROA-GLU-166 | 100 |
|  |  |  |  | PROA-TRP-168 | 0.552 | PROA-LEU-167 | 0.0121 |
|  |  |  |  |  | | PROA-HSD-172 | 0.004 |
|  |  |  |  |  |  | PROA-VAL-186 | 0.0791 |
|  |  |  |  |  |  | PROA-ASP-187 | 100 |
|  |  |  |  |  |  | PROA-ARG-188 | 0.929 |
|  |  |  |  |  |  | PROA-GLN-189 | 100 |
|  |  |  |  |  |  | PROA-GLN-192 | 0.0308 |


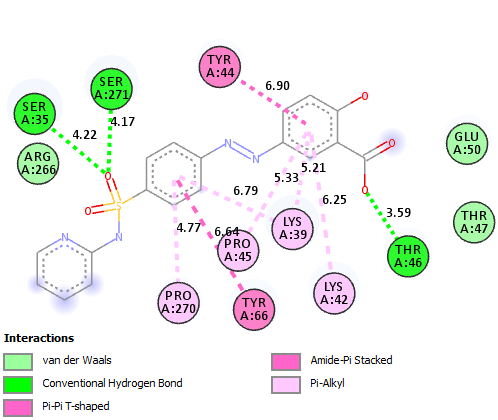
**A**
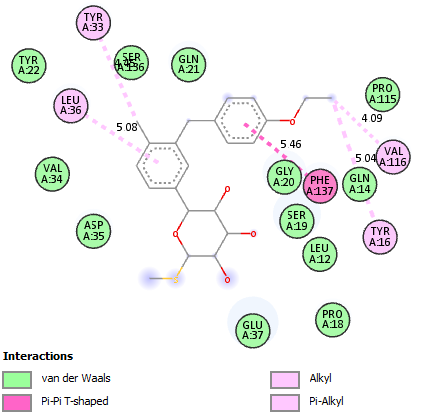
 **B**


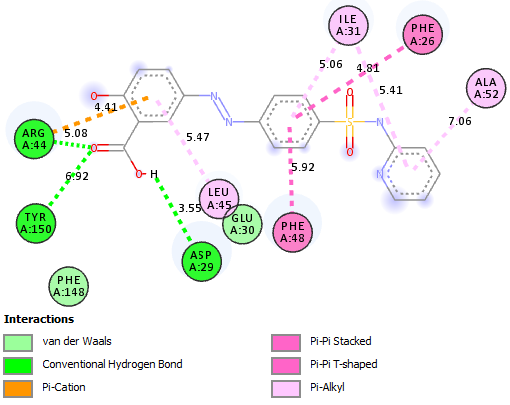
**C**
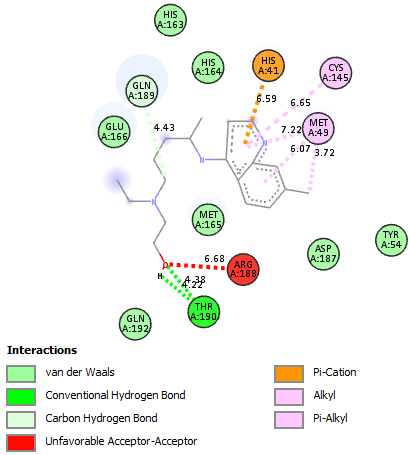
**D**Fig. S1. Molecular binding interactions (A) sulfasalazine with PTPN22 (PDB id: 4J51), (B) Sotagliflozin with type 1 diabetes receptor (PDB id: 1JK8), (C) sulfasalazine with rheumatoid arthritis receptor (PDB id: 2FSE), and (D) Hydroxychloroquine with SARS-CoV-2 receptor (PDB id: 6LU7)
