# Supplementary material for: Combining docking, molecular dynamics simulations, AD-MET pharmacokinetics properties, and MMGBSA calculations to create specialized protocols for running effective virtual screening campaigns on the autoimmune disorder and SARS-CoV-2 main protease
Source: Front Mol Biosci. 2023 Sep 1;10:1254230. doi: 10.3389/fmolb.2023.1254230 (PMC10523577; doi:10.3389/fmolb.2023.1254230)
Supplement: Supplementary file 3 [file Table2.DOCX]

**Table 2.** Computed data for the CoMFA (FFDSEL and UVEPLS) models

| Model | $R^{2}$ | $F-test$ | $SDEC$ | $Q_{loo}^{2}$ | ${SDEP}_{loo}$ | $Q_{l2o}^{2}$ | ${SDEP}_{l2o}$ | $Q_{lmo}^{2}$ | ${SDEP}_{lmo}\pm SD$ |
| --- | --- | --- | --- | --- | --- | --- | --- | --- | --- |
| CoMFA (FFDSEL) | 0.9990 | 3101.1411 | 0.0173 | 0.5548 | 0.3604 | 0.5278 | 0.3712 | 0.4721 | 0.391±0.0336 |
| CoMFA (UVEPLS) | 0.9984 | 1994.0374 | 0.0216 | 0.7033 | 0.2942 | 0.6827 | 0.3043 | 0.6305 | 0.3276±0.0224 |
