# Supplementary material for: Combining docking, molecular dynamics simulations, AD-MET pharmacokinetics properties, and MMGBSA calculations to create specialized protocols for running effective virtual screening campaigns on the autoimmune disorder and SARS-CoV-2 main protease
Source: Front Mol Biosci. 2023 Sep 1;10:1254230. doi: 10.3389/fmolb.2023.1254230 (PMC10523577; doi:10.3389/fmolb.2023.1254230)
Supplement: Supplementary file 4 [file Table5.DOCX]

**Table 5.** Calculated physicochemical properties of the top 8 compounds.

| Name | Physicochemical properties | | | | | Lipid solubility | | Water solubility | |
| --- | --- | --- | --- | --- | --- | --- | --- | --- | --- |
|  | MW | Rotatable bonds | H-bond acceptors | H-bond donors | TPSA | WLOGP | Consensus Log P | ESOL Log S | ESOL Class |
| Cpd 4 | 463.48 | 6 | 7 | 2 | 114.13 | 3.09 | 2.85 | -4.75 | Moderately soluble |
| Cpd 10 | 496.56 | 9 | 7 | 2 | 167.88 | 4.69 | 3.69 | -5.4 | Moderately soluble |
| Cpd 14 | 434.4 | 10 | 7 | 3 | 145.84 | 2.89 | 2.13 | -4.29 | Moderately soluble |
| Cpd 16 | 527.59 | 9 | 6 | 2 | 129.52 | 5 | 2.13 | -4.29 | Moderately soluble |
| Cpd 18 | 494.99 | 5 | 4 | 3 | 103.88 | 5.97 | 4.4 | -6.39 | Poorly soluble |
| Cpd 23 | 506.96 | 10 | 6 | 3 | 141.15 | 5.43 | 4.56 | -6.33 | Poorly soluble |
| Cpd 27 | 432.86 | 8 | 5 | 2 | 99.42 | 4.71 | 4.11 | -5.59 | Moderately soluble |
| Cpd 30 | 445.38 | 6 | 7 | 2 | 149.6 | 3.08 | 2.3 | -4.51 | Moderately soluble |
