# Supplementary material for: Combining docking, molecular dynamics simulations, AD-MET pharmacokinetics properties, and MMGBSA calculations to create specialized protocols for running effective virtual screening campaigns on the autoimmune disorder and SARS-CoV-2 main protease
Source: Front Mol Biosci. 2023 Sep 1;10:1254230. doi: 10.3389/fmolb.2023.1254230 (PMC10523577; doi:10.3389/fmolb.2023.1254230)
Supplement: Supplementary file 5 [file Table3.DOCX]

**Table 3.** Actual and predicted pIC50 for Autoimmune disease inhibitors of training and test set for the CoMFA (FFDSEL and UVEPLS) models.

| Cpd No. | pIC50 | FFDSEL | Residues | UVEPLS | Residues |
| --- | --- | --- | --- | --- | --- |
| 1 | 4.9821 | 5.0353 | 0.0532 | 5.0368 | 0.0547 |
| 2 | 5.0783 | 5.0828 | 0.0045 | 5.1028 | 0.0245 |
| 3 | 5.9066 | 5.8914 | -0.0152 | 5.8623 | -0.0443 |
| 4 | 4.208 | 4.2022 | -0.0058 | 4.1777 | -0.0303 |
| 5^T^ | 5.9066 | 5.6841 | -0.2225 | 5.7253 | -0.1813 |
| 6 | 5.9066 | 5.9134 | 0.0068 | 5.9163 | 0.0097 |
| 7^T^ | 5.3898 | 5.4963 | 0.1065 | 5.3324 | -0.0574 |
| 8^T^ | 5.3212 | 5.0325 | -0.2887 | 4.9931 | -0.3281 |
| 9^T^ | 4.2377 | 4.7993 | 0.5616 | 4.7328 | 0.4951 |
| 10 | 5.0256 | 5.032 | 0.0064 | 5.0316 | 0.006 |
| 1^T^ | 4.8825 | 4.9605 | 0.078 | 4.991 | 0.1085 |
| 12 | 5.6209 | 5.6058 | -0.0151 | 5.6225 | 0.0016 |
| 13 | 4.4251 | 4.4022 | -0.0229 | 4.4206 | -0.0045 |
| 14^T^ | 5.3295 | 5.1948 | -0.1347 | 5.0565 | -0.273 |
| 15 | 5.9066 | 5.9134 | 0.0068 | 5.9018 | -0.0048 |
| 16 | 5.6946 | 5.7065 | 0.0119 | 5.7177 | 0.0231 |
| 17 | 4.314 | 4.316 | 0.002 | 4.3208 | 0.0068 |
| 18 | 4.8054 | 4.8019 | -0.0035 | 4.8041 | -0.0013 |
| 19 | 4.9317 | 4.9092 | -0.0225 | 4.9552 | 0.0235 |
| 20 | 5.7964 | 5.7713 | -0.0251 | 5.7755 | -0.0209 |
| 21^T^ | 4.415 | 5.4392 | 1.0242 | 5.4792 | 1.0642 |
| 22^T^ | 5.9066 | 5.1667 | -0.7399 | 5.2997 | -0.6069 |
| 23 | 5.0665 | 5.0541 | -0.0124 | 5.0475 | -0.019 |
| 24^T^ | 5.3562 | 5.8161 | 0.4599 | 5.8035 | 0.4473 |
| 25 | 5.5599 | 5.5712 | 0.0113 | 5.5696 | 0.0097 |
| 26 | 5.1922 | 5.1893 | -0.0029 | 5.1659 | -0.0263 |
| 27 | 4.9512 | 4.9748 | 0.0236 | 4.9329 | -0.0183 |
| 28 | 5.9066 | 5.9155 | 0.0089 | 5.9257 | 0.0191 |
| 29 | 4.8247 | 4.8182 | -0.0065 | 4.8278 | 0.0031 |
| 30 | 4.8604 | 4.8719 | 0.0115 | 4.8626 | 0.0022 |
| 31 | 5.9066 | 5.8916 | -0.015 | 5.8922 | -0.0144 |

^T^ Test set compounds.
