# Supplementary material for: Combining docking, molecular dynamics simulations, AD-MET pharmacokinetics properties, and MMGBSA calculations to create specialized protocols for running effective virtual screening campaigns on the autoimmune disorder and SARS-CoV-2 main protease
Source: Front Mol Biosci. 2023 Sep 1;10:1254230. doi: 10.3389/fmolb.2023.1254230 (PMC10523577; doi:10.3389/fmolb.2023.1254230)
Supplement: Supplementary file 6 [file Table4.DOCX]

**Table 4.** Present and absent of the first hit compound against the selected targets

| Compound | PTPN22 | T1D | T1D | RA | RA | SARS |
| --- | --- | --- | --- | --- | --- | --- |
| 4 | √ | √ | √ | √ | √ | √ |
| 10 | √ | √ | - | - | √ | - |
| 14 | √ | - | - | - | - | - |
| 16 | √ | √ | √ | √ | √ | √ |
| 18 | √ | √ | √ | √ | √ | √ |
| 23 | √ | √ | √ | √ | √ | √ |
| 27 | √ | √ | - | - | - | - |
| 30 | √ | - | √ | √ | √ | √ |

NB: √ = Present; - = absent
