# Supplementary material for: Combining docking, molecular dynamics simulations, AD-MET pharmacokinetics properties, and MMGBSA calculations to create specialized protocols for running effective virtual screening campaigns on the autoimmune disorder and SARS-CoV-2 main protease
Source: Front Mol Biosci. 2023 Sep 1;10:1254230. doi: 10.3389/fmolb.2023.1254230 (PMC10523577; doi:10.3389/fmolb.2023.1254230)
Supplement: Supplementary file 7 [file Table6.DOCX]

**Table 6.** Calculated free binding energy for the complexes estimated using MM/GBSA analysis and the values of the average variation

| Parameter | PTPN22 | DT1 | RA | SARS-2 |
| --- | --- | --- | --- | --- |
| δ E(internal) | X | 10.5977 | 12.7555 | -19.6068 |
| δ E(electrostatic) + δ G(sol) | X | -17.4634 | -29.3878 | -5.8592 |
| δ E(VDW) | X | -38.2881 | -25.4669 | -38.1391 |
| δ G binding (kcal/mol) | X | -45.1538 +/- 0.3823 | -42.0993 +/- 0.4699 | -63.6051+/-0.464 |
| RMSD (Å) | 0.98 | 1.74 | 1.50 | 1.33 |
| RG (Å) | 20.19 | 18.82 | 18.70 | 22.52 |
| RMSF (Å) | 0.39 | 0.38 | 0.47 | 0.41 |
| SASA ($Å^{2}$) | 16368.53 | 12768.89 | 12794.09 | 15792.73 |
